# Mosaic Nanocrystalline Graphene Skin Empowers Highly Reversible Zn Metal Anodes

**DOI:** 10.1002/advs.202206077

**Published:** 2022-12-05

**Authors:** Xianzhong Yang, Jiaze Lv, Cai Cheng, Zixiong Shi, Jun Peng, Ziyan Chen, Xueyu Lian, Weiping Li, Yuhan Zou, Yu Zhao, Mark H. Rümmeli, Shixue Dou, Jingyu Sun

**Affiliations:** ^1^ College of Energy Soochow Institute for Energy and Materials InnovationS Light Industry Institute of Electrochemical Power Sources Key Laboratory of Advanced Carbon Materials and Wearable Energy Technologies of Jiangsu Province Soochow University Suzhou 215006 P. R. China; ^2^ School of Physics and Electronic Engineering Sichuan Normal University Chengdu 610101 P. R. China; ^3^ Center for Hybrid Nanostructures Universität Hamburg 22761 Hamburg Germany; ^4^ Beijing Graphene Institute Beijing 100095 P. R. China; ^5^ Institute for Superconducting and Electronic Materials University of Wollongong Wollongong New South Wales 2522 Australia

**Keywords:** artificial interphase layer, highly reversible, mosaic nanocrystalline graphene skin, orientational deposition, Zn anode, Zn (002) plane

## Abstract

Constructing a conductive carbon‐based artificial interphase layer (AIL) to inhibit dendritic formation and side reaction plays a pivotal role in achieving longevous Zn anodes. Distinct from the previously reported carbonaceous overlayers with singular dopants and thick foreign coatings, a new type of N/O co‐doped carbon skin with ultrathin feature (i.e., 20 nm thickness) is developed via the direct chemical vapor deposition growth over Zn foil. Throughout fine‐tuning the growth conditions, mosaic nanocrystalline graphene can be obtained, which is proven crucial to enable the orientational deposition along Zn (002), thereby inducing a planar Zn texture. Moreover, the abundant heteroatoms help reduce the solvation energy and accelerate the reaction kinetics. As a result, dendrite growth, hydrogen evolution, and side reactions are concurrently mitigated. Symmetric cell harvests durable electrochemical cycling of 3040 h at 1.0 mA cm^−2^/1.0 mAh cm^−2^ and 136 h at 30.0 mA cm^−2^/30.0 mAh cm^−2^. Assembled full battery further realizes elongated lifespans under stringent conditions of fast charging, bending operation, and low N/P ratio. This strategy opens up a new avenue for the in situ construction of conductive AIL toward pragmatic Zn anode.

## Graphical abstract

Mosaic nanocrystalline graphene layer with N/O co‐dopants and ultrathin feature directly grown over commercial Zn foil harvests a uniform Zn deposition, expecting to facilitate the pragmatic Zn metal anodes.

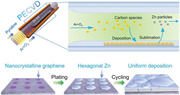

## 1 Introduction

Aqueous batteries have emerged as appealing candidates for wearable energy storage owing to the collective advantages of low cost and high safety, amongst which the rechargeable aqueous Zn‐ion battery (AZIB) has stimulated a burgeoning interest to implement mass production.^[^ ^1^, ^2^, ^3^, ^4^, ^5^ ^]^ Thereinto, Zn metal harnesses a low redox potential (−0.762 V versus standard hydrogen electrode) and a high theoretical capacity (820 mAh g^−1^, 5855 mAh cm^−3^), making it a desirable anode material in the pursuit of high‐energy battery system.^[^ ^6^, ^7^, ^8^, ^9^ ^]^ Nonetheless, the major obstacles compromising durable Zn anode lie in uncontrollable dendrite growth, adverse hydrogen evolution, and detrimental by‐product formation.^[^ ^10^, ^11^ ^]^ In further contexts, the potential practicability of Zn anode is plagued by the inferior electrochemical stability especially under the heavy‐duty operations of large current density and high Zn utilization.^[^ ^12^, ^13^ ^]^

The robust anode/electrolyte interface is a key prerequisite for realizing highly reversible Zn anodes.^[^ ^6^, ^14^ ^]^ To date, a myriad of interface engineering strategies have been explored with an emphasis on tailoring an artificial interphase layer (AIL).^[^ ^15^, ^16^, ^17^ ^]^ The Zn anodes undergo a dramatic volume change during the cycle. For instance, the average thickness variation reaches ca. 1.7 µm upon plating/stripping (1 mAh). Since an insulating AIL layer forces the reduction and deposition of Zn^2+^ to occur underneath, it has a high possibility of detaching and losing protection effects owing to the low mechanical strength and poor adhesion.^[^ ^15^ ^]^ This issue could be circumvented by constructing a conductive AIL so that Zn is deposited uniformly on its above. Nevertheless, recently reported conductive AIL derived from prevailing coating routes are prone to possess considerable thickness (i.e., >1 µm),^[^ ^17^ ^]^ which might jeopardize the overall energy density of the entire AZIB.

Carbonaceous materials, affording light‐weighted feature, structural diversity and tunable doping level, have been intensively studied in extending the lifespan of Zn anode.^[^ ^7^, ^18^, ^19^, ^20^, ^21^ ^]^ Despite fruitful achievements, Zn deposition electrochemistry mediated by carbonaceous materials remains elusive by far. Even worse, most reported Zn anodes modified by carbonaceous AIL can rarely operate under an elevated current density (>5.0 mA cm^−2^) and advanced Zn utilization conditions (depth of discharge, DOD > 40%) owing to the poor adhesion to Zn foil,^[^ ^12^ ^]^ substantially impeding the practical application. Therefore, it is essential and imperative to design an ultrathin (<50 nm) carbonaceous AIL conformally grown on commercial Zn foil targeting highly stabilized Zn anodes. Along this line, chemical vapor deposition (CVD) route, which allows the direct growth of ultrathin graphene on various substrates, is anticipated to enable the conformal graphitic formation on Zn surface and afford negligible volume/mass contribution. However, suffering from the low melting point (419.5 °C) of Zn metal, such a route involving thermal treatment has not yet done the trick in the realm of Zn anode protection.

Herein, we develop a scalable plasma‐enhanced CVD (PECVD) strategy to realize the direct growth of N/O co‐doped carbon (NOC) skin on Zn metals. A delicate balance between the sublimation of Zn and deposition of carbon is mediated, rendering NOC with varied degrees of crystal quality and electrical conductivity. Exhaustive investigations indicate that the ultrathin (20 nm) NOC skin presenting mosaic nanocrystalline graphene structure harvests the optimized protection performances, which is in favor of upholding dendrite‐free Zn deposition along the preferable (002) plane. Meanwhile, the NOC layer enables enhanced adsorption of water molecules in solvated Zn^2+^ and hence facilitates the desolvation process. Thanks to the in situ interface modulation, symmetric cell with NOC@Zn anode can sustain a durable operation at conditions of 1.0 mA cm^−2^/1.0 mAh cm^−2^ and 30.0 mA cm^−2^/30.0 mAh cm^−2^. More encouragingly, assembled full cells comprising NOC@Zn anode and vanadium oxide cathode demonstrate a prolonged lifespan under high current density, low negative‐to‐positive capacity ratio (N/P ratio), and bending conditions.

## 2 Results and Discussion

**Figure 1:**
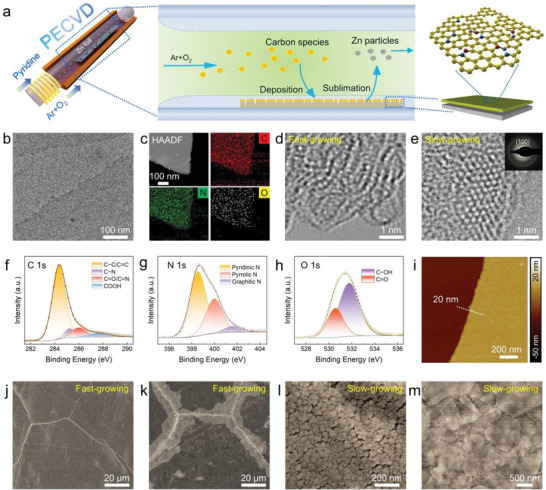
Synthesis and characterization of NOC@Zn. a) Schematic illustration of the PECVD growth of uniform NOC skin over Zn foil. b) TEM image of NOC layer. c) EDS mapping of NOC layer. HRTEM images of d) fast‐growing and e) slow‐growing NOC layer. Inset: corresponding SAED pattern. High‐resolution f) C 1s, g) N 1s, h) O 1s XPS spectra of NOC layer. i) AFM image of NOC layer. SEM image of fast‐growing NOC@Zn j) before electrodeposition and k) after electrodeposition. SEM image of slow‐growing NOC@Zn l) before electrodeposition and m) after electrodeposition.

With the consideration of the low melting point of Zn, a PECVD route^[^ ^22^ ^]^ was devised to in situ grow conformal NOC layer directly on commercial Zn foil at a temperature range of 300−400 °C. As depicted in **Figure** **1**a, pyridine was employed as the precursor to ensure the generation of N‐containing carbon species under plasma treatment (Figure S1a, Supporting Information). The growth rate of NOC layer can be accurately regulated by adjusting the flow rate of pyridine (i.e., the partial pressure). The carrier gas Ar (containing a trace amount of O_2_) can aid the transport of carbon species from upstream to Zn metal surface.^[^ ^23^, ^24^ ^]^ Impressively, the color of Zn foil rapidly turns to light brown upon reaction for 10 min, implying the successful formation of carbon coating (Figure S1b, Supporting Information). A macroscopically sized sample with a lateral dimension of 20 cm × 12 cm can be obtained by employing a 4‐inch furnace (Figure S1c, Supporting Information), holding promise for the scalable production of NOC‐skinned Zn metals in an economic fashion. Impressively, directly‐grown NOC skin affording excellent adhesion and robustness remains intact over Zn foil upon bending/twisting, which is essential for heavy‐duty operations (Figure S2, Supporting Information). In contrast, self‐assembled graphene oxide coating shows obvious disconnection off the Zn surface after the identical deformation treatment, losing its ability to protect Zn anode (Figure S3, Supporting Information). Raman spectra of NOC layers produced at different reaction temperatures were collected (Figure S4, Supporting Information), where two peaks centering at 1345 and 1596 cm^−1^ are attributed to the typical D band (defective feature) and G band (graphitic feature) signals of carbonaceous materials, respectively. The D/G intensity ratio (*I*_D_/*I*_G_) gradually decreases from 0.98 to 0.93 with the rise of growth temperature from 300 to 380 °C, indicative of improved crystallinity.^[^ ^25^ ^]^ Note that the carbon deposition is always accompanied by the Zn sublimation under a low pressure. Sublimation and melting of Zn become extremely rampant at temperatures above 400 °C, resulting in uncontrollable CVD process (Figure S5, Supporting Information). Hence, 380 °C was selected as an optimum growth temperature in our procedure.

High‐resolution aberration‐corrected transmission electron microscopy (HRTEM) was used to unravel the exact atomic structure of NOC. The TEM image at low magnification corroborates the lamellar feature of NOC layer (Figure 1b; Figure S6a, Supporting Information). Energy dispersive X‐ray spectroscopy (EDS) mapping under high‐angle annular dark field‐scanning TEM (HAADF‐STEM) mode showcases the homogeneous distribution of C, N and O elements (Figure 1c), implying the successful incorporation of nitrogen and oxygen atoms.^[^ ^26^, ^27^ ^]^ It was found that the growth rate of NOC on Zn foil exerted a profound effect on the growth quality. Because of the sublimation phenomenon, Zn foil is prone to break if the carbon deposition rate is too slow. Although fast‐growing NOC can quickly cover the Zn surface to buffer the Zn sublimation, the resulting NOC is otherwise of poor quality. As shown in HRTEM images (Figure 1d; Figure S6b, Supporting Information), the fast‐growing NOC layer comprises turbostratic domains consisting of randomly distributed carbon atoms, indicative of a fully amorphous nature.^[^ ^28^ ^]^ As for the slow‐growing NOC, the honeycomb nanocrystalline graphene domain emerges as a mosaic phase in NOC matrix (Figure 1e; Figures S6c,d and S7, Supporting Information), suggesting elevated crystallinity. Diffraction ring could be recognized in the selected area electron diffraction (SAED) pattern (Figure 1e inset), verifying the formation of graphitic nanodomains.^[^ ^29^ ^]^ In further contexts, the electrical conductivities of NOC layers obtained under different growth rates were measured (Table S1, Supporting Information). The conductivity value of fast‐growing NOC layer is lower than 5.0 × 10^−7^ S cm^−1^, showing an electrically insulative state. In contrast, slow‐growing NOC material displays a boosted electrical conductivity.

X‐ray photoelectron spectroscopy (XPS) measurement was carried out to probe the chemical composition of as‐grown NOC film. The survey spectrum bears out the existence of Zn, C, N, and O elements (Figure S8, Supporting Information). The C 1s spectrum encompasses four peaks at 284.4, 285.2, 286.1 and 287.8 eV (Figure 1f), which can be respectively assigned to C—C/C=C, C—N, C=O/C=N and —COOH bonding.^[^ ^26^ ^]^ As shown in Figure 1g, the high‐resolution N 1s spectrum can be deconvoluted into the signals of pyridinic‐N (*N*_py_, 398.5 eV), pyrrolic‐N (*N*_pr_, 400.1 eV) and graphitic‐N (*N*_gr_, 401.7 eV),^[^ ^22^, ^30^ ^]^ disclosing the presence of N dopants in NOC layer. Meanwhile, the high‐resolution O 1s spectrum was also analyzed (Figure 1h), where the two signals at 530.6 and 531.8 eV are associated with C=O and C—OH bonding. The thickness of NOC skin could be adjusted by the growth duration, which can be simply reflected by the varied color of coated Zn surface (Figure S9, Supporting Information). To obtain detailed thickness information, NOC layer was first grown on mica and then transferred on SiO_2_/Si substrate to allow atomic force microscopy (AFM) characterization. The thickness of NOC layer can be fine‐tuned, reaching ca. 20 nm upon a 10 min PECVD synthesis (Figure 1i; Figure S10, Supporting Information). Furthermore, upon the introduction of heteroatom dopants in NOC layer, the aqueous electrolyte contact angle decreases from 87° to 61° (Figure S11, Supporting Information). It is anticipated that the enhanced wettability is beneficial to facilitating the ion transport and hence reversible Zn plating/stripping.^[^ ^31^ ^]^

Representative scanning electron microscopy (SEM) observation of fast‐growing NOC@Zn demonstrates a film‐like morphology with a wealth of randomly distributed cracks (Figure 1j; Figure S12a, Supporting Information), which might originate from the uneven thermal expansion during the PECVD reaction. Apart from the cracks, the remaining regions are covered with a smooth carbon film, which is not conducive to Zn^2+^ transport (Figure S12b, Supporting Information). In turn, the Zn deposition behavior on NOC@Zn was evaluated by electroplating 0.015 mAh Zn. Impressively, Zn would merely deposit along the cracks (Figure 1k; Figure S12c,d, Supporting Information). This is mainly because the relatively dense and insulating NOC layer derived via fast growth can transport neither electrons nor Zn ions. Nevertheless, Zn foil would also show damage at a too slow growth rate (Figure S13a,b, Supporting Information). In our work, an optimized partial pressure of 8 Pa was selected for the conductive NOC growth (Figure S13c, Supporting Information). The produced surface is featured by the presence of nanopores (Figure 1l; Figure S13d, Supporting Information), which is beneficial to facilitating the transport of Zn^2+^. More encouragingly, the electro‐deposited Zn possesses a regularly hexagonal stacking structure (Figure 1m; Figure S13e,f, Supporting Information). EDS mapping results further confirmed that Zn was deposited on the upper surface of NOC layer (Figure S14, Supporting Information),^[^ ^20^ ^]^ suggesting that an electrical conductivity of 2.0 × 10^−5^ S cm^−1^ is sufficient for electron transport to ensure Zn‐ion reduction. Furthermore, side‐view SEM observation indicates the thickness of Zn foil decreases by ≈2 µm (from 100 to 98 µm) upon PECVD owing to the Zn sublimation (Figure S15, Supporting Information). Collectively, these results verify the successful preparation of conductive NOC layer affording N/O co‐doping over Zn foil by PECVD. Such an ultrathin carbon skin, which lacks long‐range order but presents mosaic nanocrystalline graphene, is anticipated to play a crucial role in guiding Zn electrodeposition.

**Figure 2:**
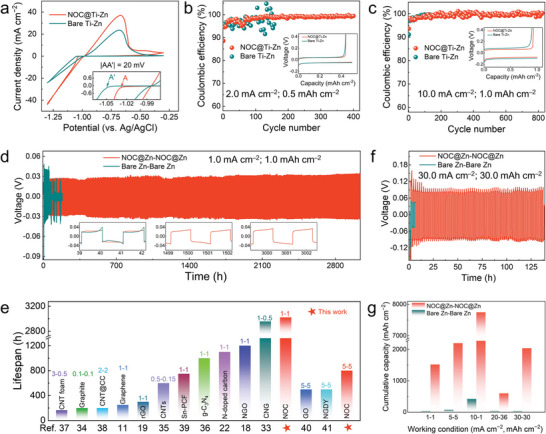
Electrochemical performances of NOC@Zn anode. a) CV curves of NOC@Ti—Zn and bare Ti—Zn cells at a scan rate of 5 mV s^−1^. CE measurement of NOC@Ti—Zn and bare Ti—Zn cells under b) 2.0 mA cm^−2^/0.5 mAh cm^−2^ and c) 10.0 mA cm^−2^/1.0 mAh cm^−2^. Inset: Corresponding voltage–capacity profiles of the first cycle. d) Cycling performances of symmetric cells at 1.0 mA cm^−2^/1.0 mAh cm^−2^. e) The comparison of cyclic reversibility between this work and previous reports. “1‐1” denotes that a current density is 1.0 mA cm^−2^ and a capacity is 1.0 mAh cm^−2^. f) Cycling performances of symmetric cells at 30.0 mA cm^−2^/30.0 mAh cm^−2^. g) Cumulative capacity of NOC@Zn and bare Zn anodes at various working conditions.

Cyclic voltammetry (CV) curves of Ti—Zn half cells were collected to probe the Zn nucleation behavior in the presence of NOC skin. As shown in **Figure** **2**a, the apparently lower Zn plating overpotential on NOC@Ti than that on bare Ti is beneficial to activating more nucleation sites, thereby promoting uniform Zn deposition. Note that the NOC@Ti—Zn cell maintains a steady cycling of over 400 cycles under 2.0 mA cm^−2^/0.5 mAh cm^−2^ (Figure 2b; Figure S16, Supporting Information), whereas the bare Ti—Zn cell can merely sustain up to 100 cycles with a drastic fluctuation of Coulombic efficiency (CE) values. More encouragingly, the NOC@Ti—Zn cell can obtain an average CE of 99.5% over 800 cycles under an elevated current density of 10.0 mA cm^−2^ (Figure 2c; Figure S17, Supporting Information), which is superior to its counterpart. The voltage hysteresis of NOC@Ti—Zn cell is far lower than that of bare Ti—Zn cell. When elevating the discharge capacity to 2.0 and 5.0 mAh cm^−2^, the NOC@Ti—Zn cell can still maintain a longevous cycling (Figure S18, Supporting Information).

Symmetric cell measurements were carried out to further elucidate the reversibility of Zn plating/stripping under the regulation of NOC skin. As shown in Figure 2d, the voltage profile of bare Zn cell gradually fluctuates and eventually short circuits after 45 h. In comparison, the NOC@Zn symmetric cell acquires a stable cycling performance over 3040 h at 1.0 mA cm^−2^/1.0 mAh cm^−2^, whose cyclic life is approximately 66 times longer than bare Zn congener. As such, the voltage hysteresis of NOC@Zn can maintain stability at ≈30 mV upon cycling, indicative of the effective inhibition of dendrite growth and by‐product formation.^[^ ^32^ ^]^ When elevating the current density to 10 mA cm^−2^ (Figure S19a, Supporting Information), the NOC@Zn symmetric cell still displays a highly stable voltage profile that sustains more than 1550 h. Meanwhile, the NOC@Zn symmetric cell harvests durable cycling performances at high capacities of 5.0 and 10.0 mAh cm^−2^ (Figure S19b,c, Supporting Information). Figure 2e and Table S2, Supporting Information, draw the performance comparisons between this work and previous reports,^[^ ^11^, ^18^, ^19^, ^22^, ^33^, ^34^, ^35^, ^36^, ^37^, ^38^, ^39^, ^40^, ^41^ ^]^ demonstrating the superior performance of NOC AIL to other carbon materials. Since the prevailing Zn metal anodes are typically compromised by shallow DOD,^[^ ^13^ ^]^ it is meaningful to augment the Zn utilization to evaluate the protective effect of NOC. In this sense, the NOC@Zn symmetric cell enables a stable cycling over 60 h under a plating/stripping capacity of 36.0 mAh cm^−2^ (Figure S20a,b, Supporting Information). The corresponding DOD of ≈63% is far higher as compared to most reported Zn anodes (Table S3, Supporting Information). Remarkably, it can also sustain a stable cycling operation over 136 h under 30.0 mA cm^−2^/30.0 mAh cm^−2^ (Figure 2f; Figure S20c, Supporting Information), outperforming most of the state‐of‐the‐art Zn electrodes under such a stringent condition. Furthermore, symmetric cell performances with different NOC thicknesses were compared. An optimized NOC thickness of 20 nm could be gained in response to enabling a prolonged cycle life (Figure S21, Supporting Information).

To gain insight into the specific roles of nitrogen and oxygen co‐doping in anode protection, the carbon film doped solely with oxygen on Zn foil (OC@Zn) was also prepared by using ethanol precursor (Figure S22, Supporting Information). It can be observed that OC@Zn has the smallest electrolyte contact angle, suggesting the positive effect of oxygen doping in improving the electrolyte wettability. Although the cyclic performance of OC@Zn has a relative improvement over the bare Zn, it is still inferior to that of NOC@Zn, indicating the leading role of nitrogen doping in improving the cyclic reversibility. In addition, the NOC@Zn symmetric cell harvests a lower voltage hysteresis than bare Zn counterpart at each current density varying from 1.0 to 40.0 mA cm^−2^ (Figure S23, Supporting Information), implying facile charge transfer kinetics under the management of NOC skin. The drastically reduced nucleation overpotential of symmetric cell can be harvested under various current densities upon the introduction of NOC (Figure S24, Supporting Information), which is beneficial to the uniform deposition of Zn. To acquire a comprehensive understanding of electrochemical performance, the calculated cumulative capacity of NOC@Zn symmetric cell can be substantially elevated to 600, 1520, 2040, 2200 and even 7750 mAh cm^−2^ under different working conditions (Figure 2g), which is superior to that of bare Zn symmetric cell. Obviously, the NOC skin can boost homogeneous Zn nucleation and growth toward highly reversible dendrite‐free Zn anode.

**Figure 3:**
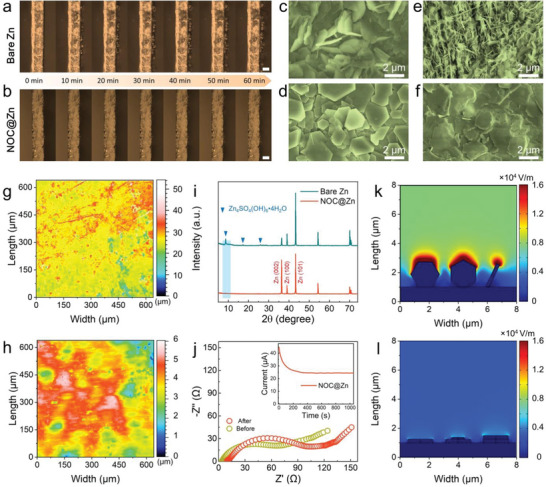
Instrumental insights into the protective effect of NOC skin. Operando optical microscopy visualization of Zn plating on a) bare Zn and b) NOC@Zn electrode at 5.0 mA cm^−2^. Scale bars: 50 µm. Top‐view SEM images of c) bare Zn and d) NOC@Zn electrodes after plating of 1.0 mAh cm^−2^ with a current density of 10.0 mA cm^−2^. Top‐view SEM images of e) bare Zn and f) NOC@Zn electrodes after 100 cycles under 10.0 mA cm^−2^/1.0 mAh cm^−2^. Optical surface profilometry images of g) bare Zn and h) NOC@Zn after 100 cycles under 10.0 mA cm^−2^/1.0 mAh cm^−2^. i) XRD patterns of bare Zn and NOC@Zn electrodes after 100 cycles under 10.0 mA cm^−2^/1.0 mAh cm^−2^. j) Nyquist plots of NOC@Zn symmetric cell before and after polarization. Inset: Current evolution of NOC@Zn symmetric cell under polarization of 10 mV. Simulated electric field distributions on k) bare Zn and l) NOC@Zn electrodes.

To shed light on the protective mechanism of such ultrathin NOC skin, exhaustive characterizations in combination with electrochemical measurements were carried out. In situ optical microscopy was first employed to visualize Zn plating behavior with a current density of 5.0 mA cm^−2^ (Figure S25, Supporting Information). After electrodeposition for 10 min, a multitude of protrusions randomly appear on the surface of bare Zn, which become increasingly obvious during electrodeposition (**Figure** **3**a; Video S1, Supporting Information). NOC@Zn electrode otherwise maintains a smooth surface morphology without discernible dendrites (Figure 3b; Video S2, Supporting Information). The detailed morphologies of Zn plating were disclosed by SEM to further elucidate the impact of NOC conformal coating on mitigating the dendrite. As depicted in Figure 3c and Figure S26a, Supporting Information, Zn flakes with high dip angles are randomly deposited on bare Zn, whilst hexagonal Zn plates on NOC@Zn are parallel to the substrate and exhibit a highly ordered orientation (Figure 3d; Figure S26b, Supporting Information). Upon plating/stripping for 100 cycles at 10.0 mA cm^−2^/1.0 mAh cm^−2^, rampant dendrites and pits can be observed on the surface of bare Zn foil (Figure 3e; Figure S26c, Supporting Information). In stark contrast, the NOC@Zn electrode enables a dense and flat Zn deposition morphology (Figure 3f; Figure S26d, Supporting Information). Accordingly, the stripped bare Zn electrode (Figure S26e, Supporting Information) is full of ravines owing to the uneven stripping process, while the NOC@Zn (Figure S26f, Supporting Information) displays a uniform morphology. Optical surface profilometry imaging further shows the NOC@Zn electrode displays a far smaller surface height difference (6 µm, Figure 3h) as compared to the bare Zn (50 µm, Figure 3g) after electrochemical cycling, in good agreement with side‐view SEM observation (Figure S27, Supporting Information). Therefore, the NOC skin can facilitate the homogeneous Zn plating/stripping and suppress the dendrite growth even under high current densities (Figure S28, Supporting Information).

X‐ray diffraction (XRD) was employed to examine the crystal structure of electrodes after Zn deposition. The (002) intensity on NOC@Zn is greatly enhanced in comparison with bare Zn (Figure 3i), implying a highly oriented deposition. The guided (002)‐oriented Zn deposition via NOC skin contributes to a dendrite‐free morphology of NOC@Zn anode. With respect to the bare Zn electrode, a noticeable peak at 8.6° can be recognized in the XRD pattern, which is ascribed to the formation of detrimental by‐product namely Zn_4_SO_4_(OH)_6_·4H_2_O.^[^ ^6^ ^]^ Nonetheless, this diffraction peak can rarely be observed for NOC@Zn electrode, suggesting that side reactions can be effectively mitigated by the NOC. In addition, the NOC@Zn symmetric cell possesses a higher transference number^[^ ^42^ ^]^ than bare Zn (Figure 3j; Figure S29, Supporting Information), implying that the NOC skin can promote Zn^2+^ transport and hence facilitate homogenous Zn deposition. The orientation of deposited Zn plates plays a vital role in influencing the electric field distribution of Zn anode. In this case, finite element simulation based on COMSOL Multiphysics was carried out to explore the electric field distributions (Figure S30, Supporting Information). With respect to the bare Zn, the electrical field at the corner of vertical plates is drastically enhanced (Figure 3k), which would accordingly trigger uneven Zn deposition. On the contrary, the horizontal Zn plates endow the NOC@Zn electrode with homogeneously distributed electrical field (Figure 3l). These results uncover that the horizontal deposition of Zn plates can aid to homogenize the electric field distribution, thereby further contributing to a uniform ion flux and ultimately promoting the reversibility of Zn anode.^[^ ^20^ ^]^

**Figure 4:**
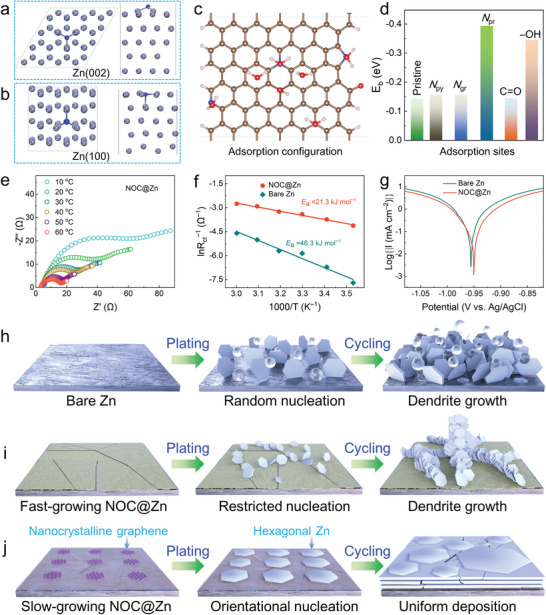
Theoretical calculation and electrochemical analysis. Optimized adsorption models of N atom on a) Zn (002) and b) Zn (100) planes. The gray and blue balls denote the zinc and nitrogen atoms, respectively. c) The adsorption configuration of water molecule on NOC layer. The brown, red, blue and white balls indicate carbon, oxygen, nitrogen and hydrogen atom, respectively. d) The binding energy of water molecule on different adsorption sites. e) Nyquist plots at different temperatures for NOC@Zn. f) Arrhenius curves and comparison of activation energies of bare Zn and NOC@Zn. g) Linear polarization curves presenting the corrosion on bare Zn and NOC@Zn. Schematic diagrams of h) bare Zn, i) fast‐growing NOC@Zn, and j) slow‐growing NOC@Zn electrodes upon electrochemical cycling.

The planar Zn deposition morphology is of paramount significance to the construction of highly durable NOC@Zn anode. The slow‐growing NOC layer possesses an optimized electrical conductivity of ≈2.0 × 10^−5^ S cm^−1^, which supports the Zn‐ion reduction and subsequent Zn deposition on its forefront surface. As reported,^[^ ^43^ ^]^ the slight mismatch (7%) of hexagonal lattice between graphene and Zn (002) plane could ensure a heteroepitaxial deposition behavior when graphene was used as current collector. In our case, as a conductive AIL on Zn, the NOC skin affording mosaic nanocrystalline graphene can also induce orientational deposition, which might stem from two aspects: i) guided deposition on graphene domains; and ii) heteroatom doping effect. In response, density functional theory (DFT) calculations were performed to reveal the adsorption mechanism of N and O atoms on different crystal planes including Zn (002), Zn (100), and Zn (101) (**Figure** **4**a,b; Figure S31, Supporting Information). This is anticipated to further help exploring the role of NOC skin in manipulating Zn nucleation. Note that the free N/O atoms and fully relaxed crystal surface were selected as initial sites for structural optimization. Our DFT calculation indicates that the adsorption energy of N/O atom on Zn (002) is slightly lower than that on Zn (100) and Zn (101) (Table S4, Supporting Information), which might be caused by the higher stability of Zn (002) facet.^[^ ^44^, ^45^ ^]^ More intriguingly, the optimized adsorption configurations imply that the N/O atom is prone to bury into the Zn (100) or Zn (101) surface in contrast to the adsorption scenario on Zn (002), which would cause the erosion of Zn(100)/Zn(101).^[^ ^46^ ^]^ Consequently, the moderate binding energy of N/O atom on Zn (002) is beneficial to guiding preferential nucleation along Zn (002) plane at the initial stage of electrodeposition. Meanwhile, the unstable embedded configurations manifest that the Zn nuclei can barely grow along Zn (100) and Zn (101) during the plating process. In other words, the incorporated N/O atoms in the carbon skeleton could induce the nucleation along Zn (002). Collectively, the NOC skin promotes Zn (002)‐dominated nucleation and growth behaviors, thereby mitigating the dendrite formation and ensuring the excellent stability of the Zn plating/stripping process.

The solvation sheath of Zn^2+^ (Zn(H_2_O)_6_^2+^) is also a key to regulating the interfacial redox reaction.^[^ ^47^, ^48^ ^]^ It is established that Zn^2+^ can be reduced and deposited on the electrode only after removing the solvation sheath.^[^ ^41^ ^]^ To elucidate the effect of NOC layer on modulating the solvation structure in the charge‐transfer process, the adsorption energy between H_2_O and NOC was accordingly calculated. The optimized adsorption configurations of H_2_O with six adsorption sites are depicted (Figure 4c; Figure S32, Supporting Information). The relatively low binding energy (−0.14 eV) between pristine graphene and H_2_O molecule would give rise to a weak adsorption effect (Figure 4d). With the incorporation of O doping, the increased binding energy (−0.142 eV for C=O and −0.34 eV for C—OH) indicates an enhanced capture capability for H_2_O. With respect to the three types of N doping, the *N*_pr_ harvests the highest binding energy of −0.39 eV. These simulation results suggest that Zn(H_2_O)_6_^2+^ is prone to interact with NOC skin with the main aid of *N*_pr_ and —OH sites, thereby facilitating the desolvation process.

The electrochemical impedance spectra (EIS) at varied temperatures were collected to unravel the desolvation effect of NOC skin. It is evident that the charge‐transfer resistance (*R*_ct_, Table S5, Supporting Information) is drastically reduced with the assistance of NOC layer (Figure 4e; Figure S33, Supporting Information). As illustrated in Figure 4f, the quantitatively derived activation energy^[^ ^6^, ^49^ ^]^ (*E*_a_) of NOC@Zn symmetric cell (21.3 kJ mol^−1^) is considerably lower than that of bare Zn (46.3 kJ mol^−1^). The high ionic conductivity of the NOC layer is beneficial to its Zn^2+^ transport (Figure S34, Supporting Information). The Zn^2+^ diffusion dynamics tested by chronoamperometry imply the introduction of NOC layer can effectively inhibit the 2D diffusion,^[^ ^49^ ^]^ thereby promoting homogeneous deposition (Figure S35, Supporting Information). The linear polarization test was carried out to explore the corrosion of Zn anode (Figure 4g). In comparison with the bare Zn anode, the corrosion potential of NOC@Zn obtains a positive shift (from −0.956 to −0.950 V). In addition, the declined corrosion current by ≈302.3 µA cm^−2^ also suggests the effective restriction of corrosion effect with the presence of NOC skin. Therefore, the NOC skin avoids the direct contact between Zn anode and electrolyte, which further inhibits the side reaction and corrosion effect.^[^ ^20^ ^]^

Taken together, the plating and cycling process on bare Zn and NOC@Zn anodes are schematically illustrated (Figure 4h–j). In the case of bare Zn anode, a wealth of randomly distributed Zn plates, pits and H_2_ bubbles aggregate on the surface after repeated plating/stripping, thereby resulting in a curtailed lifespan (Figure 4h). As for the fast‐growing NOC AIL, Zn only nucleates and deposits over crack sites because of the dense and insulative carbon layer, which still leads to dendrite formation and rapid short‐circuit of the battery (Figure 4i). In contrast, the slow‐growing conductive NOC skin can mitigate dendrite growth and stabilize Zn anode via two mechanisms (Figure 4j). For one thing, it can guide the oriented nucleation on crystalline graphene mosaic and enable the in‐parallel deposition with respect to the substrate. For another, it can significantly boost the interfacial redox kinetics by decreasing desolvation energy, thus ultimately achieving a prolonged cycle life.

**Figure 5:**
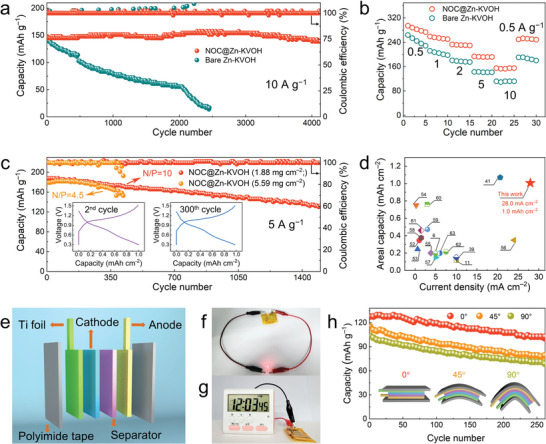
Electrochemical performances of Zn‐ion full cells. a) Cycling performance at 10.0 A g^−1^ for 4000 cycles. b) Rate performance. c) Cycling performance at N/P ratio of 10.0 and 4.5. d) Comparison of current density and areal capacity between this work and previous reports. e) Schematic illustration of the flexible transparent battery. Digital photos showing the working states of flexible NOC@Zn—KVOH cells in series to power. f) A LED indicator and g) a timer. h) Cycling performances of the flexible AZIB under different bending angles. Insets: diagrams of flexible batteries under three bending angles.

To envisage the potential usage of NOC@Zn anode in practical devices, AZIB full cells comprising a KV_12_O_30‐y_·nH_2_O (KVOH)^[^ ^6^, ^50^ ^]^ cathode and a NOC@Zn anode were assembled. The NOC@Zn—KVOH cell delivers an initial capacity of 148.1 mAh g^−1^ and maintains at 141.9 mAh g^−‍1^ with a capacity retention of 96% after 4000 cycles at 10.0 A g^−‍1^ (**Figure** **5**a). In stark contrast, the bare Zn—KVOH cell undergoes a rapid capacity decay due to the rampant dendrite growth and side reactions.^[^ ^41^, ^51^ ^]^ Rate performance measurements indicate that NOC@Zn—KVOH cell harvests an advanced capacity at each current density as compared to bare Zn—KVOH, especially at the high rates (Figure 5b). Such an excellent rate capability could be attributed to the robust anode/electrolyte interface. Nyquist plots reveal that the NOC@Zn—KVOH cell exhibits lower charge‐transfer resistance and faster ion diffusion kinetics in comparison with the bare Zn—KVOH counterpart (Figure S36, Supporting Information).

As a matter of fact, low loading cathodes (≈1.0 mg cm^−2^) and thick Zn anodes (≥100 µm) were ubiquitously employed in previously reported AZIB systems.^[^ ^3^, ^43^ ^]^ Consequently, the N/P ratio is typically higher than 50, which greatly hinders the improvement of energy density. It is imperative and meaningful to increase the mass loading of cathodes and reduce the thickness of Zn anodes. To corroborate the outstanding electrochemical performance of the as‐prepared NOC@Zn anodes in AZIB full cells, high‐loading cathodes (5.59 mg cm^−2^) and thin Zn foils (10 µm) were employed. As aforementioned, the inevitable sublimation of Zn occurs in the PECVD process and hence the thickness of Zn foil can be decreased by 2 µm, which stands for a capacity of 4.68 mAh cm^−2^. As shown in Figure 5c, the cyclic durability degrades in response to the reduction of N/P ratio. Encouragingly, the NOC@Zn—KVOH cell can sustain a stable operation of over 400 cycles at 5.0 A g^−1^ at a low N/P ratio of 4.5. Furthermore, the average charging time of NOC@Zn—KVOH full cell is as short as 2.2 min at 5 A g^−1^, which is expected to meet the needs of pragmatic fast‐charging scenarios (Figure S37, Supporting Information). Figure 5d draws a comparison of current density and areal capacity based on Zn anode between this work and previous reports,^[^ ^6^, ^11^, ^39^, ^41^, ^52^, ^53^, ^54^, ^55^, ^56^, ^57^, ^58^, ^59^, ^60^, ^61^, ^62^, ^63^ ^]^ manifesting the superiority of NOC@Zn anode (Table S6, Supporting Information).

More impressively, NOC@Zn anode harnessing mechanical robustness enables facile construction of bendable full cells in pursuit of flexible energy storage applications. As depicted in Figure 5e, KVOH cathode and NOC@Zn anode are separated by glass fiber and encapsulated by polyimide tape.^[^ ^64^ ^]^ Two full cells in series can easily power a light‐emitting diode (LED) indicator (Figure 5f) or an electronic timer (Figure 5g), showcasing the promising application potential. Benefiting from its mechanical robustness, such a flexible full cell harvesting a low charge‐transfer resistance delivers stable operation for over 250 cycles under different bending states (Figure 5h; Figure S38, Supporting Information). Notably, the bendable full cell achieves a favorable capacity retention of 68% with a bending angle of 90°. Typically, the capacity of bendable full cells decays much faster than that of coin‐type cells, this is because the lack of tight interfacial contact in bendable full cell might prevent the smooth carrier transportation during battery operation. Moreover, the local electric field intensity might become inhomogeneous during bending, which would induce massive Zn dendrite growth in flexible batteries.^[^ ^65^ ^]^ Figure S39, Supporting Information, further demonstrates the extended‐cycling performance of NOC@Zn—KVOH soft‐packing battery with an N/P ratio of 9. These evaluations collectively substantiate that the NOC skin can inhibit parasitic reactions at the anode/electrolyte interface and guide uniform Zn deposition, ultimately in favor of elevating the stability and durability of practical AZIB devices.

## 3 Conclusions

High‐performance NOC@Zn anode has been developed throughout the direct growth of N/O co‐doped mosaic nanocrystalline graphene layer on commercial Zn foil using PECVD. The growth rate are precisely regulated to endow the NOC layer with an electrical conductivity of 2.0 × 10^−5^ S cm^−1^, ensuring the deposition of Zn on its upper surface. As‐prepared ultrathin NOC layer can not only enable dendrite‐free deposition by guiding preferential growth along Zn (002) plane, but also accelerate the kinetics of Zn^2+^ deposition via reducing desolvation energy, thereby mitigating H_2_ evolution and side reactions. As a result, symmetric cell based on NOC@Zn electrodes demonstrates a low voltage hysteresis and outstanding cyclic stability at all testing current densities/capacities, even under harsh conditions (30.0 mA cm^−2^/30.0 mAh cm^−2^; 20.0 mA cm^−2^/36.0 mAh cm^−2^). A cumulative capacity as high as 7750 mAh cm^−2^ can be achieved when cycled at 10.0 mA cm^−2^, representing one of the advanced performances. More encouragingly, full batteries equipped with NOC@Zn anodes deliver remarkable cycling stability under high rate, low N/P ratio, and bending conditions. Our NOC@Zn anodes affording versatility and simplicity might promote the commercialization of Zn metal batteries.

## Conflict of Interest

The authors declare no conflict of interest.

## Supporting information

Supporting Information

Supplemental Video 1

Supplemental Video 2

## Data Availability

The data that support the findings of this study are available from the corresponding author upon reasonable request.
