# Supplementary material for: Mosaic Nanocrystalline Graphene Skin Empowers Highly Reversible Zn Metal Anodes
Source: Adv Sci (Weinh). 2022 Dec 5;10(4):2206077. doi: 10.1002/advs.202206077 (PMC9896044; doi:10.1002/advs.202206077)
Supplement: Supplementary file 1 — Supporting Information [file ADVS-10-2206077-s002.pdf]

ADVANCED SCIENCE
Open Access

Supporting Information

for *Adv. Sci.*, DOI 10.1002/advs.202206077

Mosaic Nanocrystalline Graphene Skin Empowers Highly Reversible Zn Metal Anodes

*Xianzhong Yang, Jiaze Lv, Cai Cheng, Zixiong Shi, Jun Peng, Ziyan Chen, Xueyu Lian, Weiping Li, Yuhan Zou, Yu Zhao, Mark H. Rümmeli, Shixue Dou and Jingyu Sun**

Supporting Information

Mosaic Nanocrystalline Graphene Skin Empowers Highly Reversible Zn Metal Anodes

*Xianzhong Yang, Jiaze Lv, Cai Cheng, Zixiong Shi, Jun Peng, Ziyan Chen, Xueyu Lian, Weiping Li, Yuhan Zou, Yu Zhao, Mark H. Rümmeli, Shixue Dou, and Jingyu Sun**

Experimental Section

Synthesis of NOC@Zn

NOC@Zn was synthesized throughout a direct PECVD route. The pure Zn foil (thickness: 100 or 10 μm) was thoroughly cleaned with ethanol and water in order to remove impurities prior to use. The substrate was subsequently placed at the center of the furnace. The CVD system was pumped with a base pressure of 2 Pa and purged with a mixed carrier gas consisting of 0.2 standard cubic centimeters per minute (sccm) O_2 and 20 sccm Ar. Upon reaching to 380 °C within 5 min, the plasma (80 W) was turned on to allow the growth of 10 min with pyridine as precursor. After the PECVD reaction, the furnace was cooled down to room temperature under Ar. In our system, the flow of pyridine is simply controlled through a valve. With the passage of pyridine, the vacuum of furnace tube will decrease. The flow rate of pyridine can be indirectly reflected by the rise of pressure, thus controlling deposition rate of carbon species.

Preparation of KVOH cathodes

The cathode[1] were fabricated by mixing active material ($KV_{12}O_{30-y}\cdot nH_2O$, KVOH), conductive carbon (Super P) and polyvinylidene fluoride (PVDF) binder with a mass ratio of 7:2:1. The as-prepared slurry was uniformly dispersed in N-Methyl-2-Pyrrolidone (NMP) solvent, which was dropped onto Ti foil. The thus-fabricated electrodes were dried in a vacuum oven under 80 ℃ for 12 h to remove the residual solvent. The mass loading of active materials is 1.0~5.6 mg cm^{-2}.

Characterization

The morphologies of prepared samples were inspected by SEM (Hitachi, SU-8010). The as-grown NOC film was firstly transferred on copper mesh using a polymer-assisted transfer technology. [2] Then the detailed structure, selected area electron diffraction and elemental maps of samples were recorded on TEM (FEI, Titan Themis Cubed G2 300; 80–300 kV). The confocal Raman spectrometer (LabRAM HR Evolution) was used to acquire the Raman spectra. To analyze the surface chemistry of samples, XPS measurements were carried out by the Escalab 250Xi Spectrophotometer. XRD patterns were collected on the Bruker D8 Advance Diffractometer. The contact angle was measured using Contact Angle System SL200KS (Solon Tech. (Shanghai) Inc., China). The optical surface profilometry images were captured on Leica DCM8 microsystems.

Electrochemical tests

Three types of CR2032 coin cells were assembled to investigate the electrochemical performances, including Zn–Zn symmetric cell, Ti–Zn asymmetric cell, and Zn–KVOH full cell. As-prepared electrodes were cut into circle discs. The cells were assembled with the commercial glass fiber as separator (Whatman^{TM}) and 2 M $ZnSO_4$ as electrolyte. For the flexible full cells, the cathode, anode and glass fiber separator were all cut into rectangles, with the size of 3 cm × 2 cm, 3 cm × 2 cm, and 4 cm × 3 cm respectively. The KVOH cathode, separator and NOC@Zn anode were sealed by polyimide tape. Then the flexible cell was tightly compacted to ensure there were no bubbles. Current–time curves and EIS were collected on CHI660E electrochemical workstation CHI660E. Galvanostatic charge/discharge, rate, and cycling measurements were performed on the Neware battery-testing instrument. The linear-sweep voltammograms (LSV) were recorded with a potential ranging from –1.15 to –0.75 V (vs. Ag/AgCl) at a scan rate of 2 mV s^{-1}.

Theoretical calculations

The geometry optimization and adsorption energy of N/O atoms are obtained based on first-principles plane wave calculations within density functional theory as implemented in the Vienna ab-initio simulation package (VASP).[3-5] The projector augmented-wave method[6] and Perdew-Burke-Ernzerhof exchange-correlation functional[7] are used. A cutoff energy of 400 eV for the plane-wave basis set and a Monkhorst-Pack mesh[8] of 4×4×1 for the Brillouin zone integration are employed for Zn (002), Zn (100) and Zn (101) slabs relaxation and self-consistent calculations. Zn (002), Zn (100) and Zn (101) slabs with periodically repeating (4×4) unit cell by 5 layers was constructed for the N/O atom adsorption. The lower two layers of atoms are fixed to maintain the same with bulk structure. The thickness of vacuum layer was set to 17 Å. For H_2O adsorption, the Monkhorst-Pack mesh of 5×1×1 for the Brillouin zone integration is employed for N-graphene ribbon relaxation and self-consistent calculations. All the structures are fully relaxed by conjugate gradient method until the maximum Hellmann-Feynman force acting on each atom is less than 0.01 eV/Å. In our calculation, the Grimme's D3 dispersion correction method is used[9]. The adsorption energy was calculated by the formula: $E_{ads} = E_{total} - E_{slab} - E_{zn}$, where E_{total}, E_{slab} and E_{Zn} are the total energy with/without the adsorption of Zn atom and atomic energy of Zn, respectively.

Electric field simulation

Finite element analysis conducted by COMSOL Multiphysics software was adapted to compare the electric field distribution at the interface between Zn hexagonal plates on the electrode and electrolyte. According to the two distinguished states of Zn hexagonal plates in

the SEM images, two simplified three-dimensional models were established. In the models, one cuboid (8 μm length × 8 μm width × 7 μm height) represents the 2 M $ZnSO_4$ electrolyte with conductivity of 5 S m^{-1}, and the other cuboid (8 μm length × 8 μm width × 1 μm height) below the electrolyte represents Zn electrode. Regular hexagon sheets with different sizes were used to present the Zn hexagonal sheets produced during the charge and discharge process. The bigger one, with a base edge of 1 μm and a thickness of 0.25 μm, was twice as the size of the small one. By changing the position state of the Zn sheets, the electric field distribution in different Zn sheet positions and stacking states can be further studied. The electrical conductivity of Zn electrode and Zn sheets was set as 1.67×10^{7} S m^{-1}. The experimentally determined voltage hysteresis were employed as the cathodic potentials: the model (bare Zn) was 48 mV, while the model (NOC@Zn) was 22 mV. The anodic potential was fixed as a constant of zero.

- **Supporting Figures**

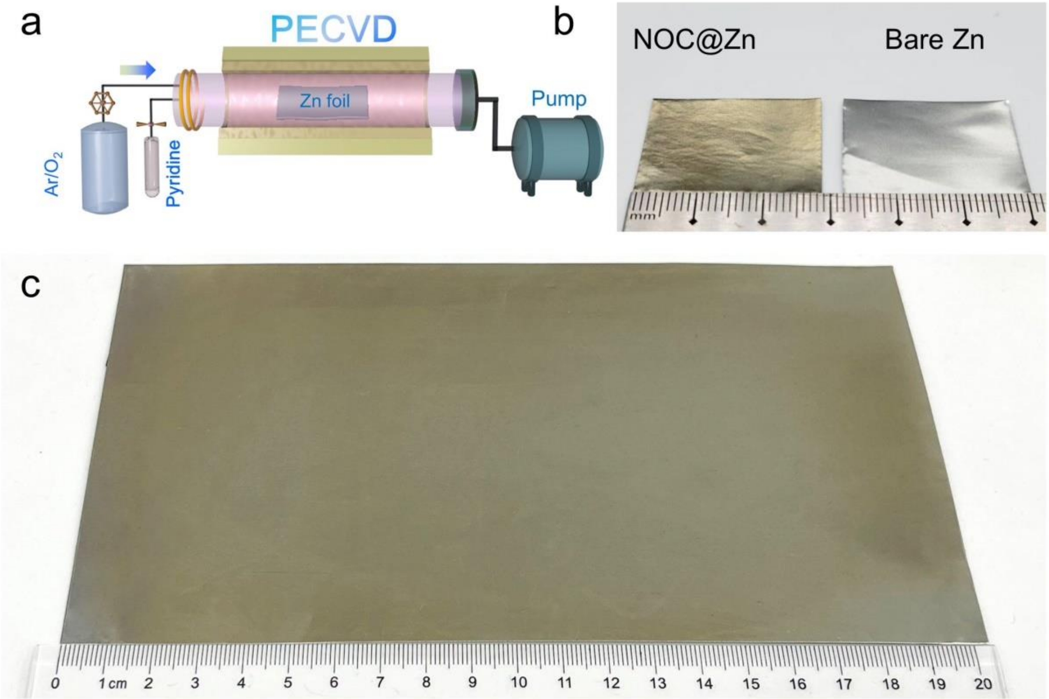

Figure S1. a) Schematic illustration of the PECVD growth of N/O co-doped nanocrystalline graphene layer on Zn foil (NOC@Zn). b) Digital photograph of NOC@Zn foil grown in a 1-inch quartz reactor and bare Zn foil. c) Digital photograph showing NOC@Zn foil grown in a 4-inch quartz reactor.

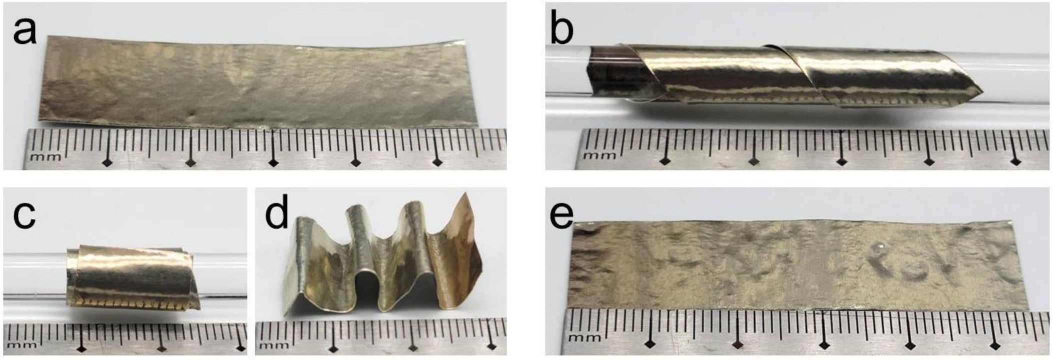

Figure S2. a) Digital photograph of as-grown NOC@Zn. Digital photograph of the b) twisting, c) rolling, and d) folding processes for NOC@Zn. e) Digital photograph of the NOC@Zn after twisting, rolling and folding processes. The NOC protective layer does not detach from the substrate upon these operations.

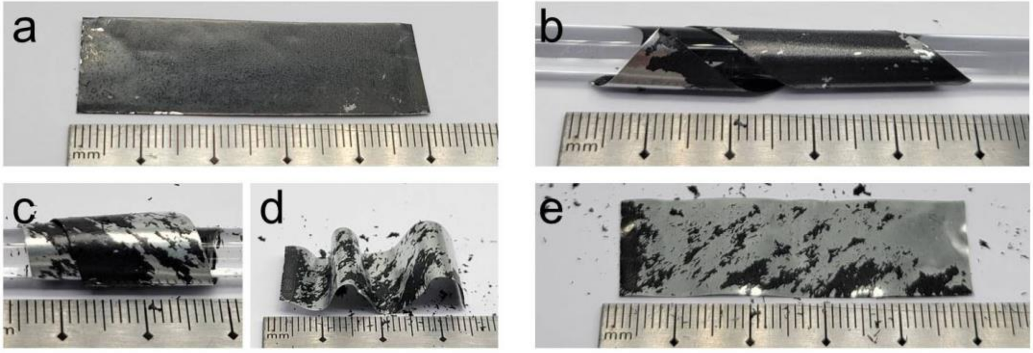

Figure S3. a) Digital photograph of self-assembled reduced graphene oxide on Zn foil (rGO@Zn). Digital photograph of b) twisting, c) rolling, and d) folding processes for rGO@Zn. e) Digital photograph of the rGO@Zn foil after twisting, rolling and folding processes. Graphene oxide was synthesized from graphite flakes by a modified Hummer's method.[10] To obtain rGO@Zn, bare Zn foil was first soaked in the GO solution for self-assembly and then dried thoroughly in an oven.[11] Because of weak adhesion, the vast majority of rGO falls off from the Zn foil.

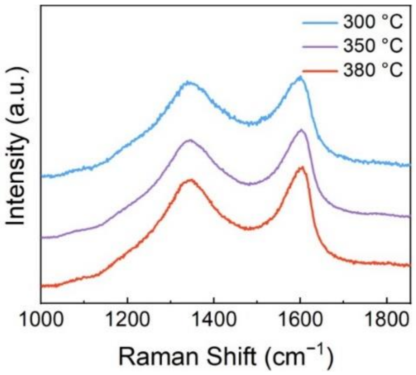

Figure S4. Raman spectra of NOC@Zn grown at different temperatures.

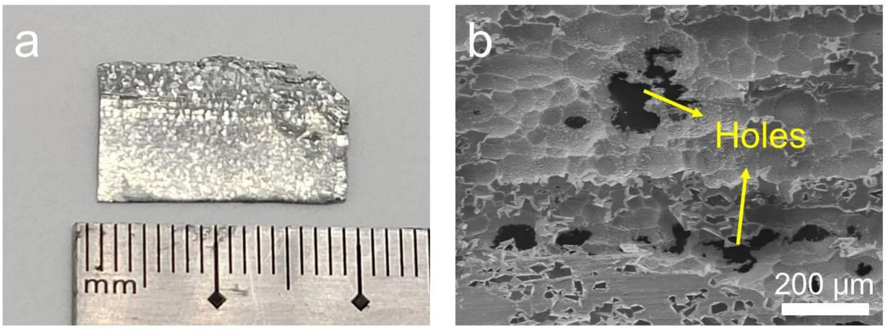

Figure S5. a) Digital photograph showing a NOC@Zn foil grown at 400 °C. b) Top-view SEM image of NOC@Zn grown at 400 °C. The surface of Zn foil would be undulating with plenty of holes when the temperature exceeded 400 °C.

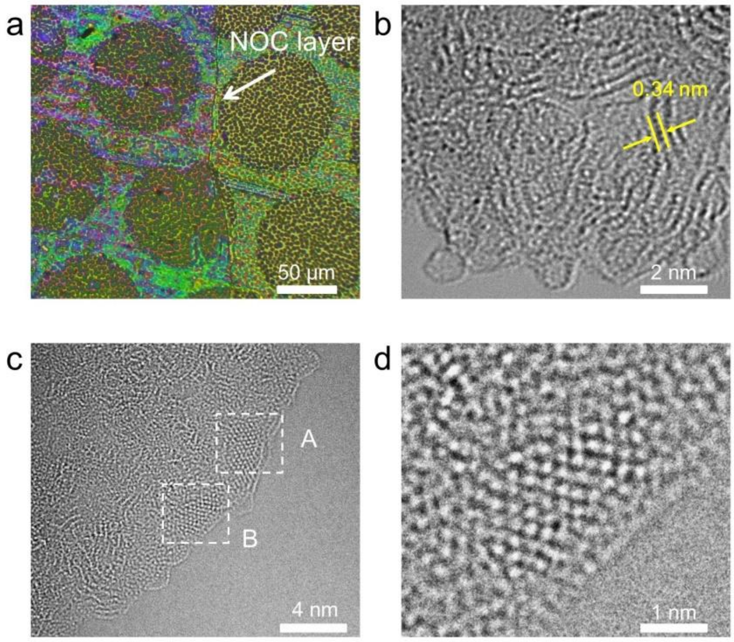

Figure S6. a) OM image of the transferred NOC layer on copper grid. b) HRTEM image of fast-growing NOC skin. c) HRTEM image of slow-growing NOC skin. d) Enlarged image of area B in c). The enlarged image of area A is shown in Figure 1e.

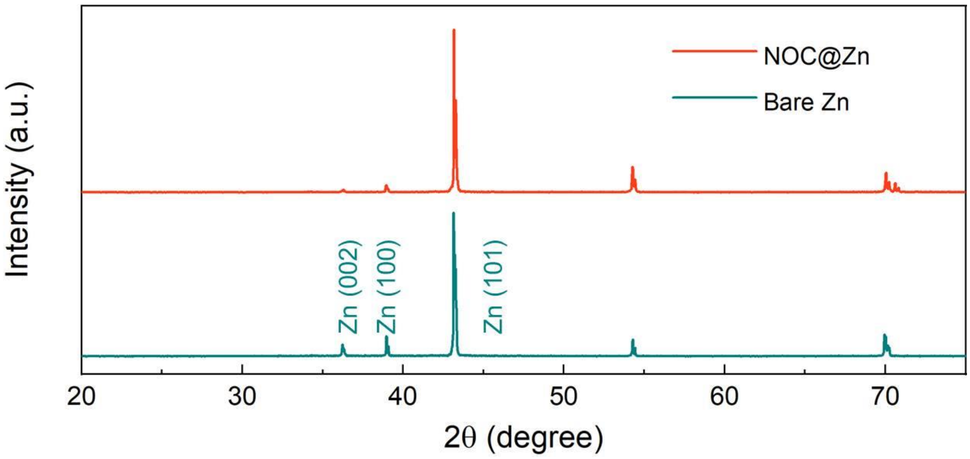

Figure S7. XRD patterns of bare Zn and NOC@Zn.

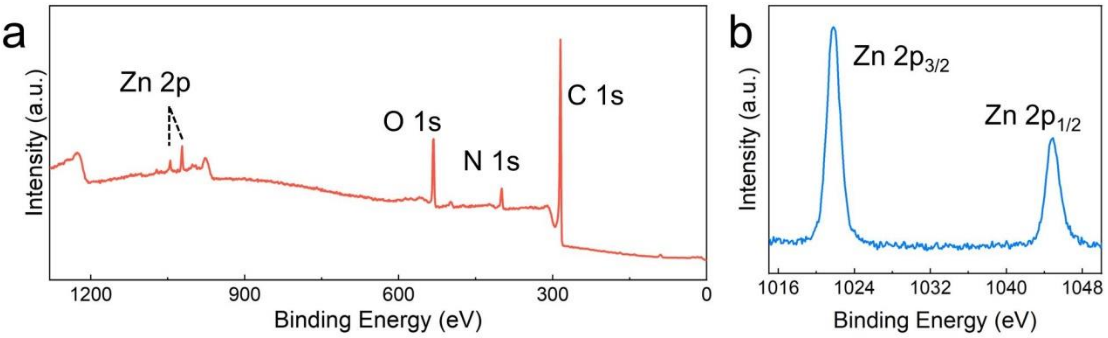

Figure S8. a) XPS survey spectrum of NOC@Zn. b) High-resolution XPS Zn 2*p* spectrum. The C, N, and O content was estimated to be 81.7, 6.1, and 12.2 at.%, respectively.

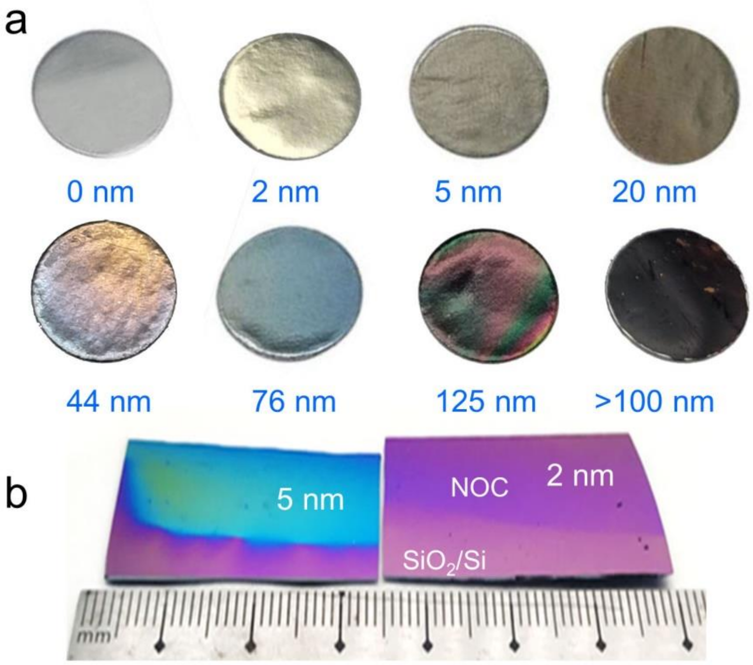

Figure S9. a) Digital photograph of NOC@Zn with different NOC thicknesses. b) Digital photograph of NOC layer on 300 nm SiO_2/Si substrate. In essence, our NOC is a two-dimensional material. The color of 2D materials is dependent on the substrate and thickness. It is the result of reflection and transmission of light, which follows the Fresnel law. Along this line, in order to show the optical contrast of NOC layer more vividly, NOC layers with different thicknesses were directly grown on SiO_2/Si substrate, where the evolution of color with thickness can be clearly identified.

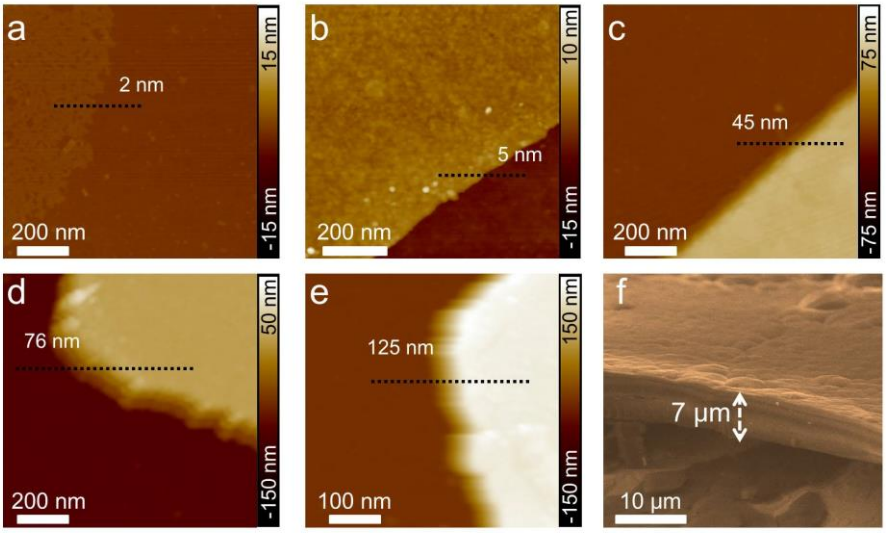

Figure S10. AFM images of the NOC layers with showing a thickness of a) 2 nm, b) 5 nm, c) 45 nm, d) 76 nm, e) 125 nm. f) Side-view SEM image of NOC@Zn with an NOC film thickness of 7 μm.

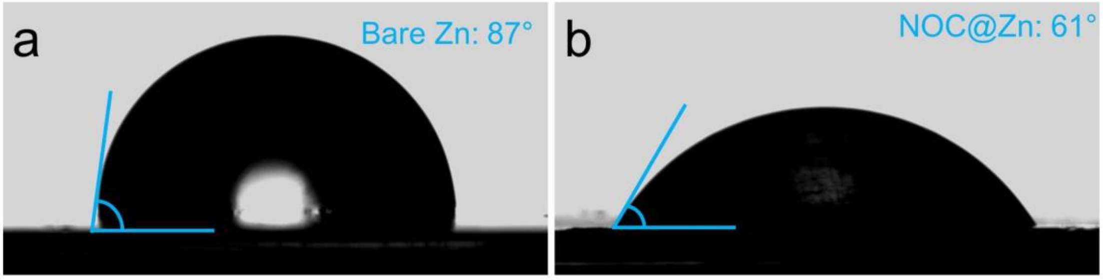

Figure S11. Contact angles of $ZnSO_4$ electrolyte on a) bare Zn foil and b) NOC@Zn.

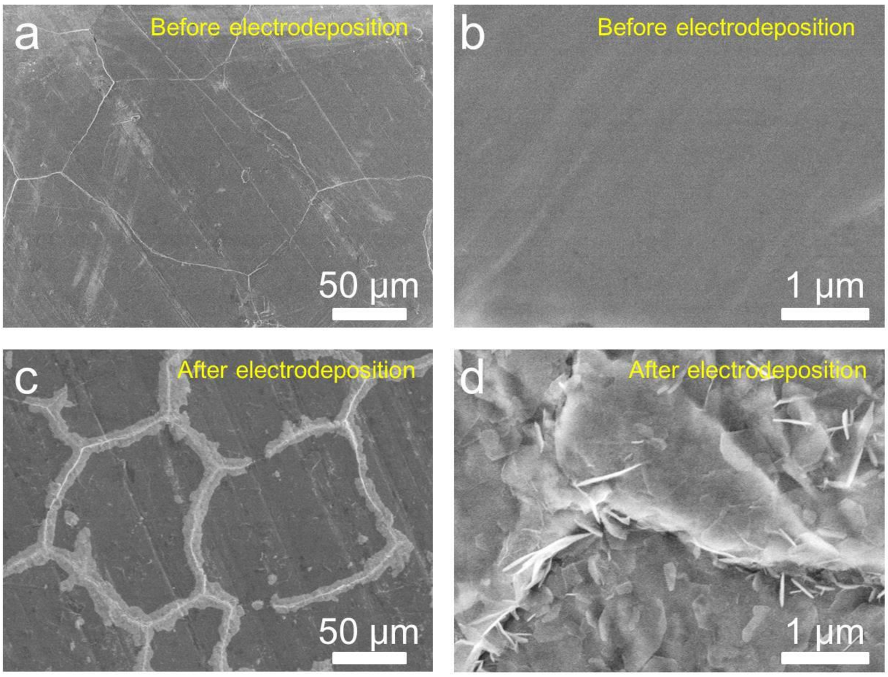

Figure S12. a) SEM image of fast-growing NOC@Zn. b) Enlarged view for a). c) SEM image showing NOC@Zn (fast-growing) after Zn plating of 0.015 mAh cm^{-2}. d) Enlarged view for c).

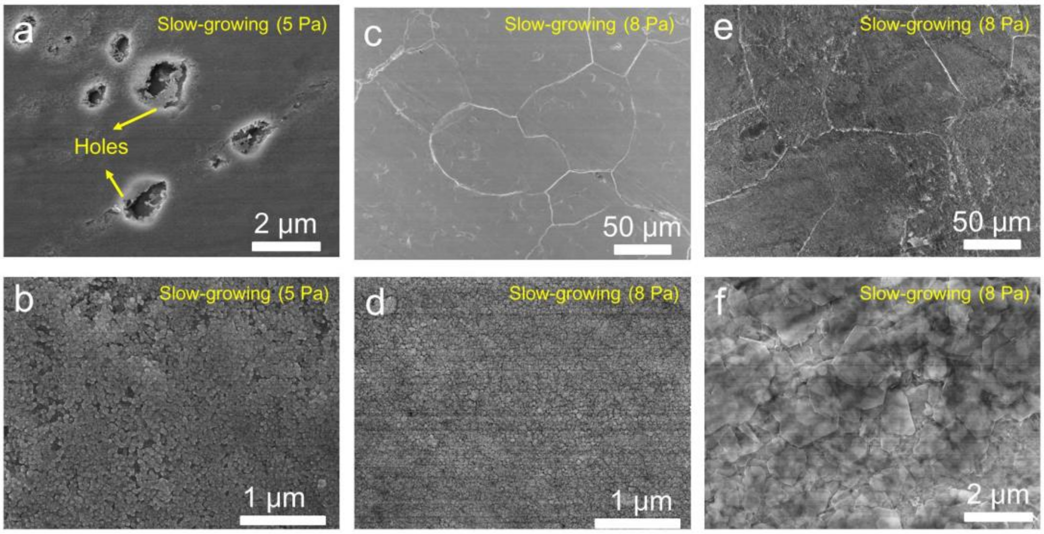

Figure S13. a) SEM image of slow-growing NOC@Zn with the pressure rise of 5 Pa. b) Enlarged image of a). c) SEM image of slow-growing NOC@Zn with the pressure rise of 8 Pa. d) Enlarged image of c). e) SEM image of NOC@Zn (slow-growing) after Zn plating of 0.015 mAh cm^{-2}. f) Enlarged image of e). If the carbon deposition rate was too slow, the Zn foil would be damaged owing to the sublimation effect.

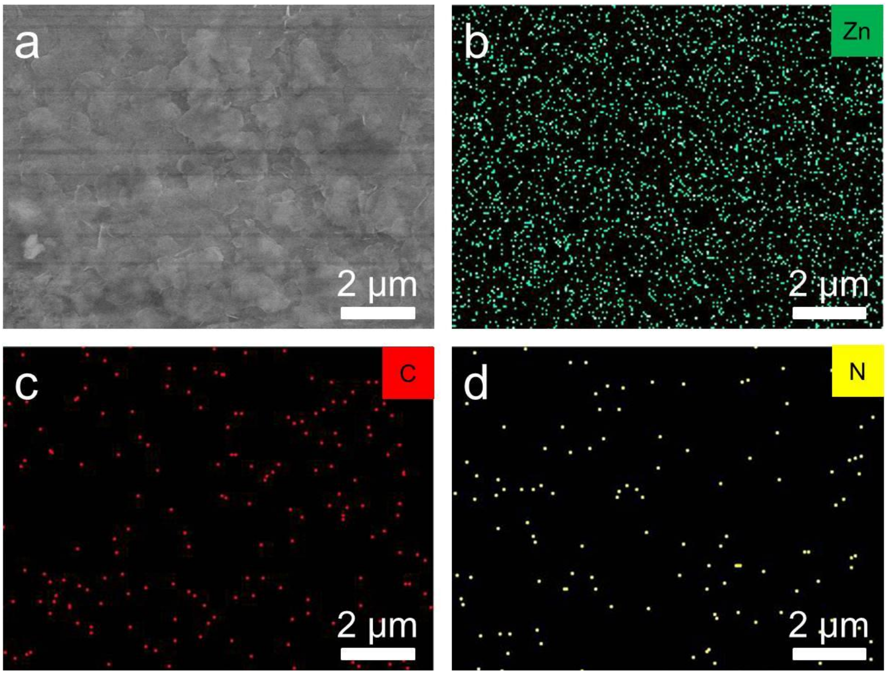

Figure S14. a) SEM image of NOC@Zn (slow-growing) after Zn plating of 0.015 mAh cm^{-2}. EDS maps of plated NOC@Zn for b) Zn, c) C, and d) N elements.

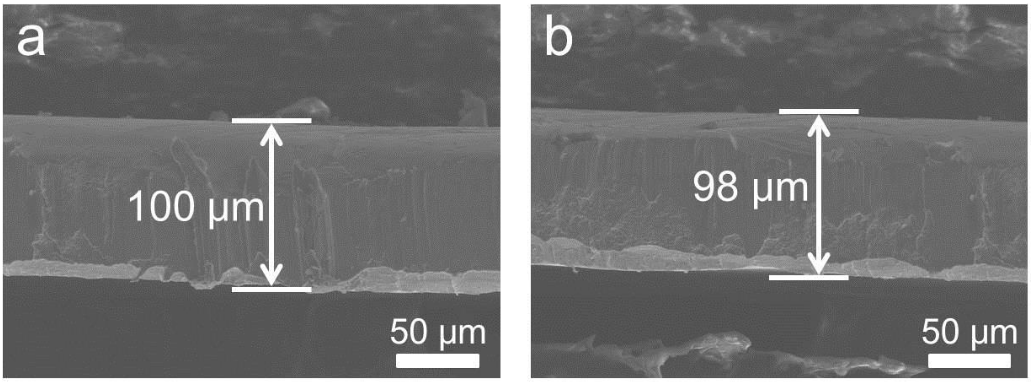

Figure S15: a) Side-view SEM image of bare Zn. b) Side-view SEM image of NOC@Zn.

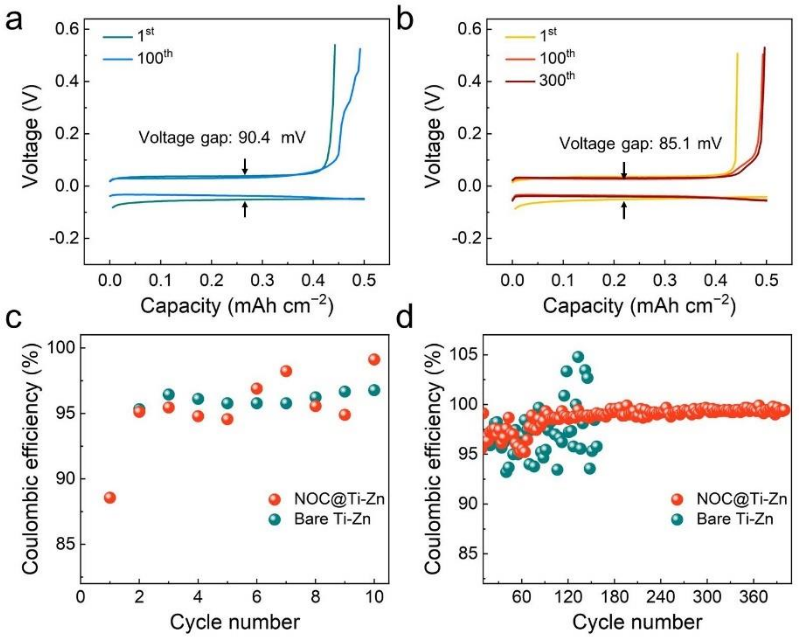

Figure S16. Charge-discharge voltage profiles of a) bare Ti–Zn cell and b) NOC coated Ti–Zn cell at the 1st, 100th, and 300th cycles under 2.0 mA cm^{-2}/0.5 mAh cm^{-2}. The enlarged images of Coulombic efficiencies shown in Figure 2c: c) 1st-10th and d) 10th-400th cycles.

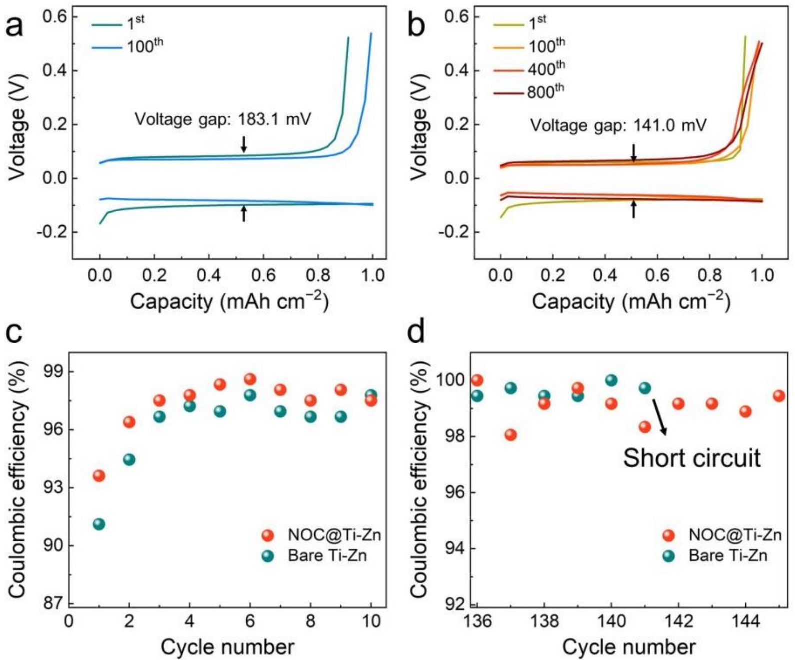

Figure S17. Charge-discharge voltage profiles of a) bare Ti–Zn cell and b) NOC coated Ti–Zn cell at the 1st, 100th, 400th and 800th cycles under 10.0 mA cm^{-2}/1.0 mAh cm^{-2}. The enlarged images of Coulombic efficiencies shown in Figure 2c: c) 1st-10th and d) 136th-145th cycles.

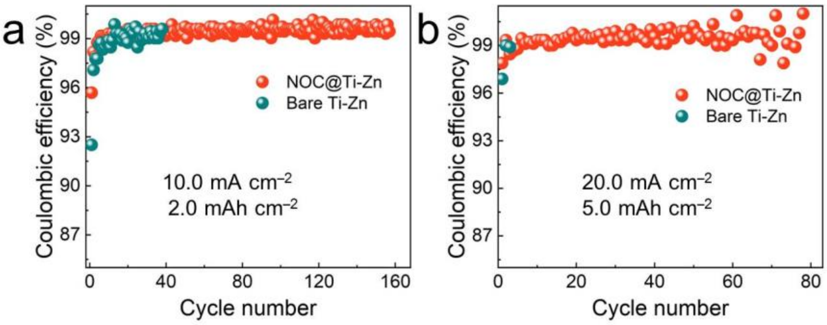

Figure S18. CE measurement of NOC@Ti–Zn and bare Ti–Zn cells under a) 10.0 mA cm^{-2}/2.0 mAh cm^{-2} and b) 20.0 mA cm^{-2}/5.0 mAh cm^{-2}. The lifespan of the CE test decreases with increasing current density and capacity. The bare Ti-Zn cell only harvests 3 cycles at 20.0 mA cm^{-2}/5.0 mAh cm^{-2} due to rampant dendrite growth, while the NOC@Ti-Zn can cycle stably over 75 cycles.

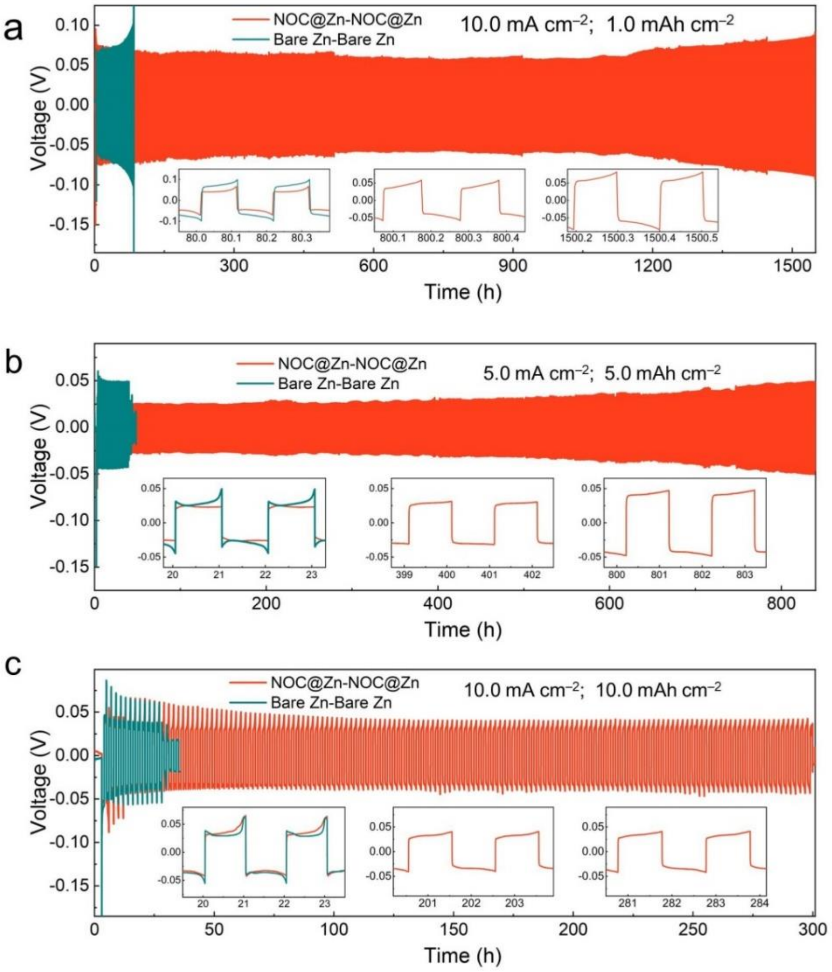

Figure S19. Long-term galvanostatic cycling of NOC@Zn and bare Zn symmetric cells at a) 10.0 mA cm^{-2} /1.0 mAh cm^{-2}, b) 5.0 mA cm^{-2} /5.0 mAh cm^{-2}, and c) 10.0 mA cm^{-2} /10.0 mAh cm^{-2}. Insets: Detailed voltage profiles at specific cycling time.

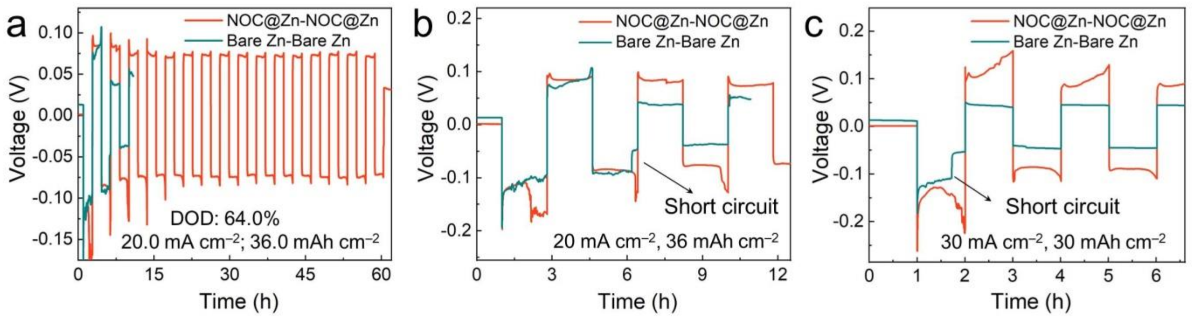

Figure S20. a) Cycling performances of symmetric cells at 20.0 mA cm^{-2}/36.0 mAh cm^{-2}. b) Enlarged image of the initial cycle in a). c) Enlarged image of the initial cycle in Figure 2f.

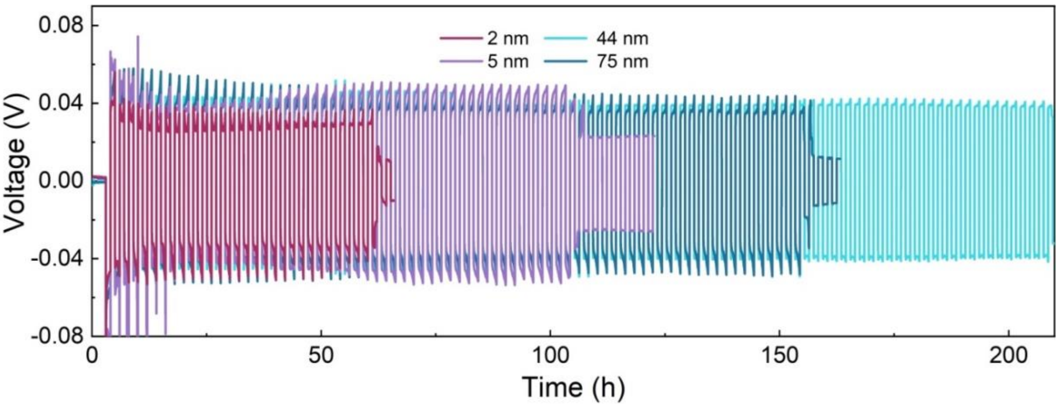

Figure S21. Long-term galvanostatic cycling of NOC@Zn symmetric cells at 10.0 mA cm^{-2}/10.0 mAh cm^{-2}. The thickness of the NOC skin is 2 nm, 5 nm, 44 nm and 76 nm, respectively. When the thickness is about 20 nm, Zn can grow on the NOC layer along (002) plane owing to proper electrical conductivity, and Zn^{2+} can be stripped and come out throughout the pores, thus ensuring a stable cycling. If the NOC layer is too thin (< 10 nm), the electrode may not be wholly covered due to its non-uniformity, where dendrites would grow. The transport of ions and electrons would deteriorate if the thickness is thick enough (> 44 nm), the lifespan of a symmetric cell is therefore reduced.

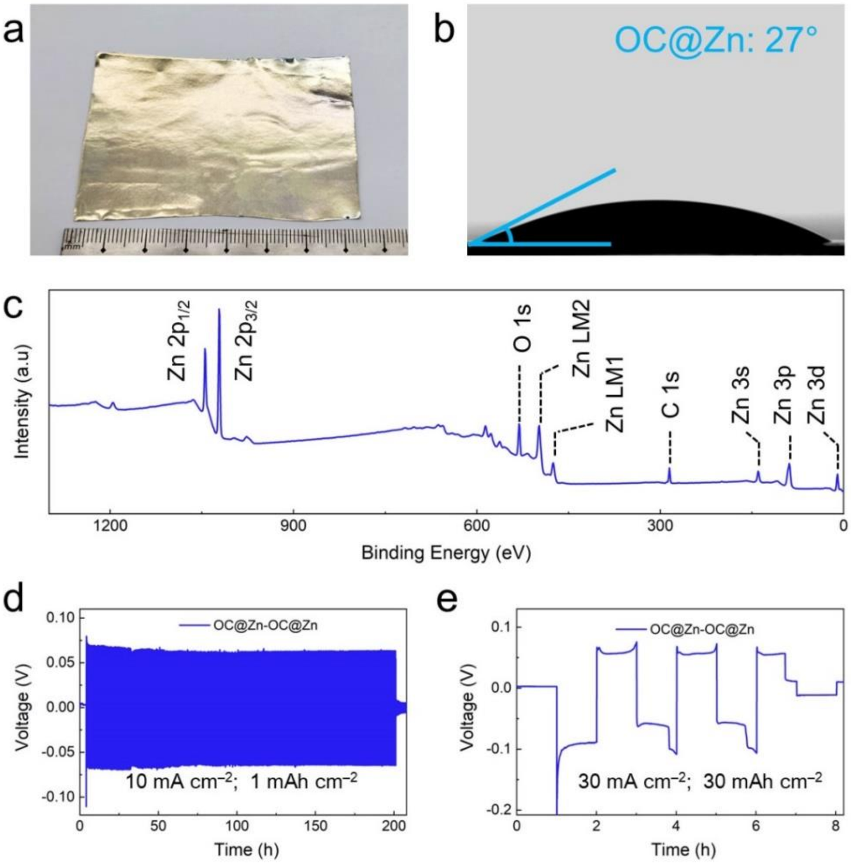

Figure S22. a) Photograph of OC@Zn foil grown by ethanol precursor. b) Contact angle of $ZnSO_4$ electrolyte on OC@Zn foil. c) XPS survey spectrum of OC@Zn. Long-term galvanostatic cycling of OC@Zn symmetric cells at a current density of d) 10.0 mA cm^{-2}/1.0 mAh cm^{-2} and e) 30.0 mA cm^{-2} /30.0 mAh cm^{-2}.

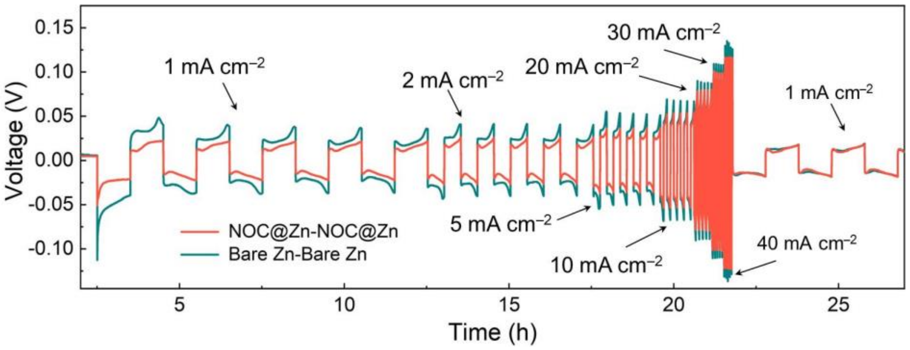

Figure S23. Rate performances of NOC@Zn and bare Zn symmetric cells at various current densities under a capacity of 1.0 mAh cm^{-2}.

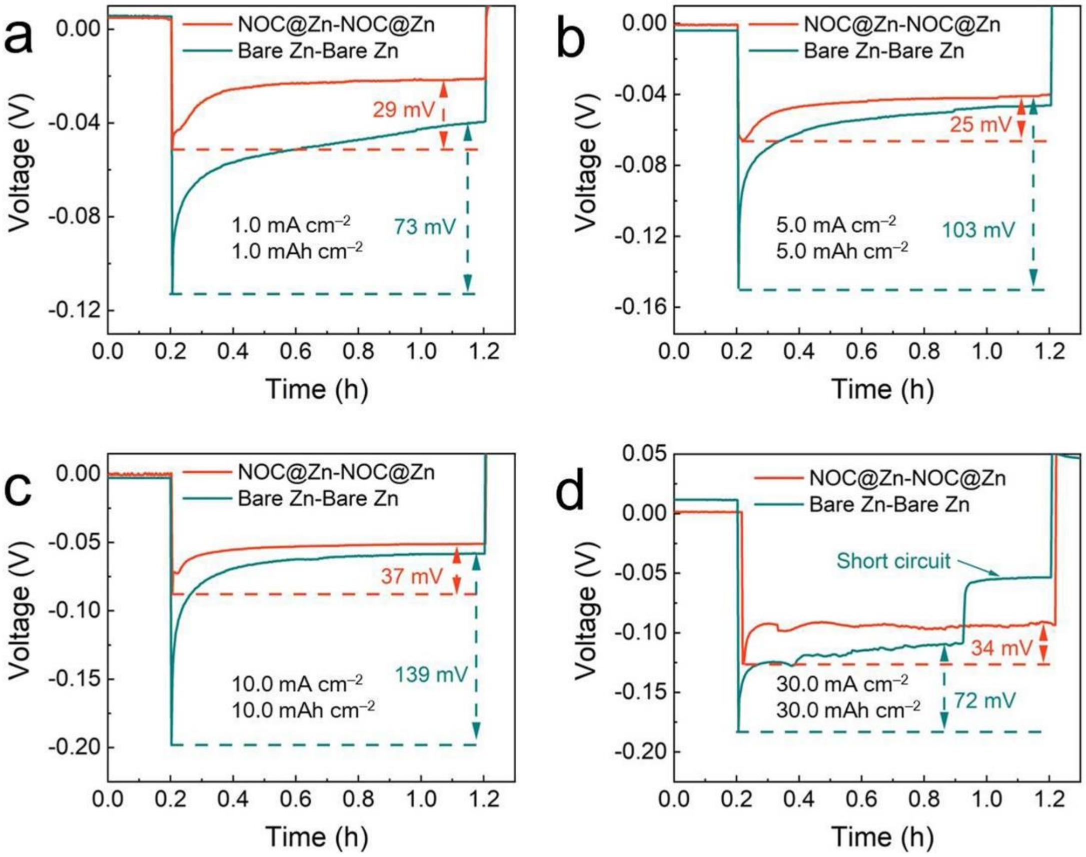

Figure S24. Nucleation overpotential for bare Zn and NOC@Zn symmetric cells at different current densities and areal capacities: a) 1.0 mA cm^{-2}, 1.0 mAh cm^{-2}; b) 5.0 mA cm^{-2}, 5.0 mAh cm^{-2}; c) 10.0 mA cm^{-2}, 10.0mAh cm^{-2}; and d) 30.0 mA cm^{-2}, 30.0 mAh cm^{-2}.

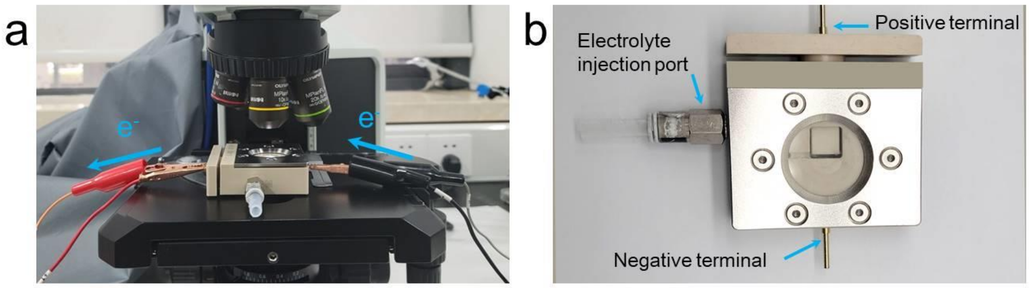

Figure S25. a) Digital image of the *in-situ* optical microscopy system for studying the electrodeposition processes of Zn anodes. b) Digital image of the transparent cell for *in-situ* optical visualization. The positive and negative terminals are connected to Neware testing system.

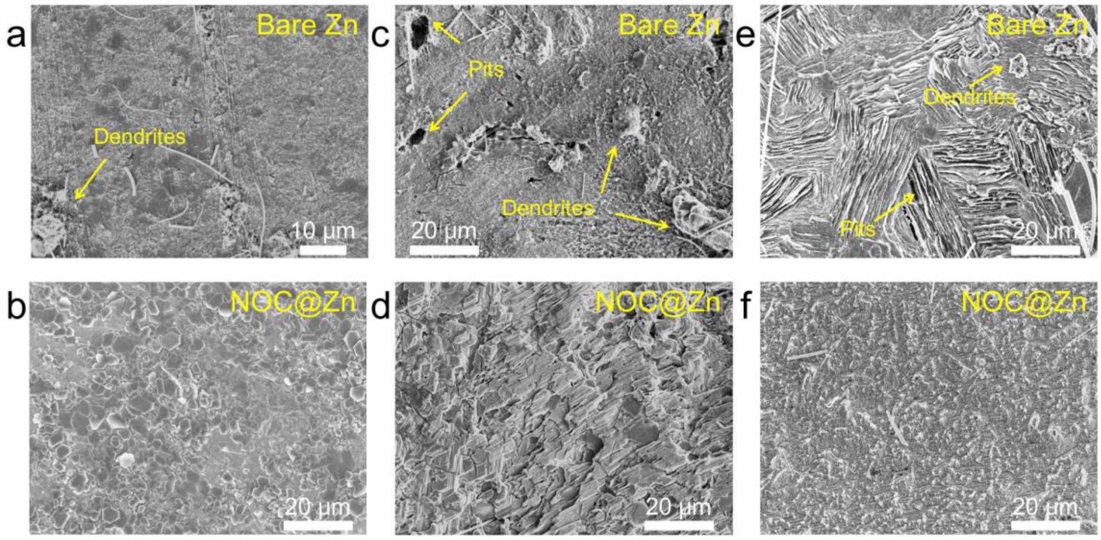

Figure S26. Top-view SEM images of a) bare Zn and b) NOC@Zn electrodes after plating of 1.0 mAh cm^{-2} with a current density of 10.0 mA cm^{-2}. Top-view SEM images of electrodes after 100 cycles under 10.0 mA cm^{-2}/1.0 mAh cm^{-2}: c) Plated bare Zn, d) stripped bare Zn, e) plated NOC@Zn, and f) stripped NOC@Zn.

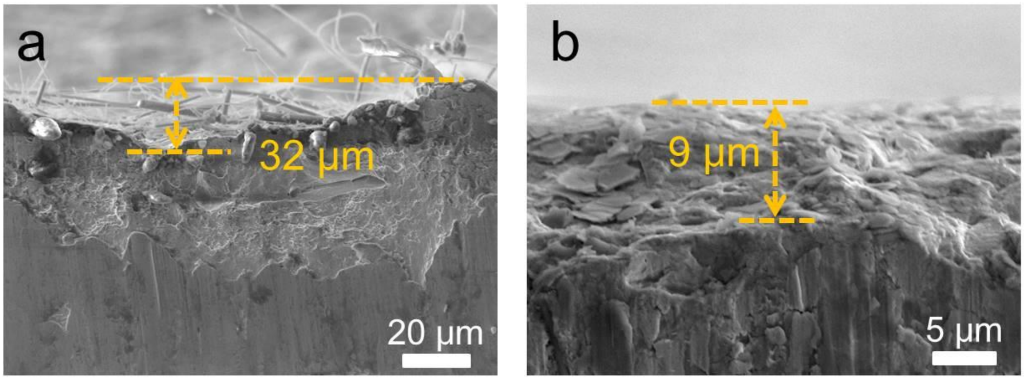

Figure S27. Side-view SEM images of a) bare Zn and b) NOC@Zn electrodes after 100 cycles under 10.0 mA cm^{-2}/1.0 mAh cm^{-2}.

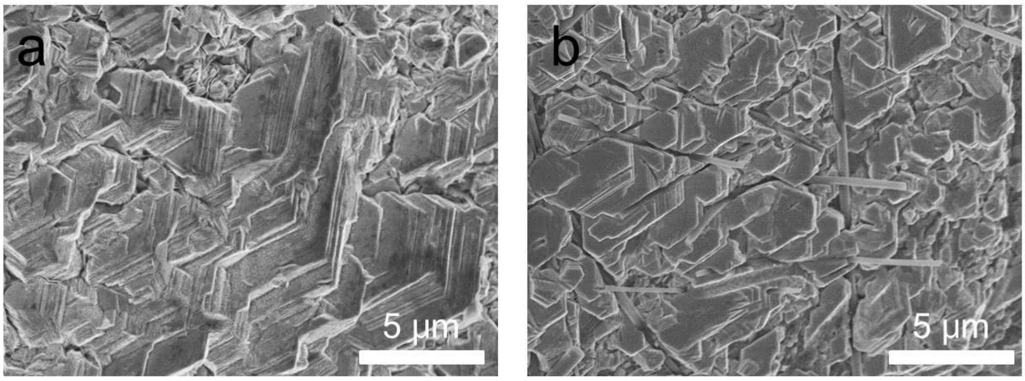

Figure S28. Top-view SEM images of NOC@Zn electrodes after plating of 1.0 mAh cm^{-2} with a current density of a) 20.0 mA cm^{-2} and b) 30.0 mA cm^{-2}.

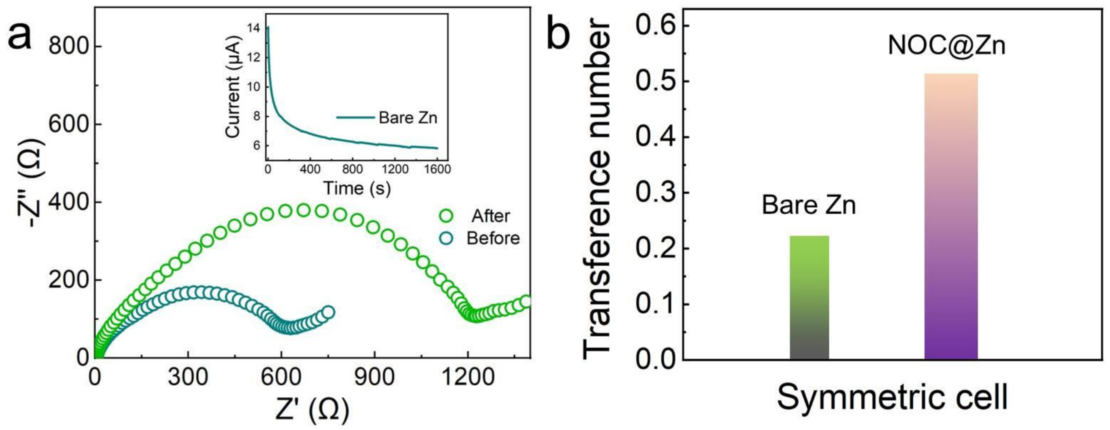

Figure S29. a) Nyquist plots of bare Zn symmetric cell before and after polarization. Inset: Current evolution of bare Zn symmetric cell under polarization of 10 mV. b) Calculated transference number of bare Zn and NOC@Zn symmetric cell. The Zn^{2+} transfer number can be deduced from the equation[12] $T_{Zn^{2+}}=\frac{I_s(\Delta V-I_0R_0)}{I_0(\Delta V-I_sR_s)}$, where I_0/I_s and R_0/R_s are the initial/steady current and interfacial resistance, respectively. ΔV was set as 10 mV. The calculated transfer number for bare Zn and NOC@Zn symmetric cell is 0.22, 0.51, respectively.

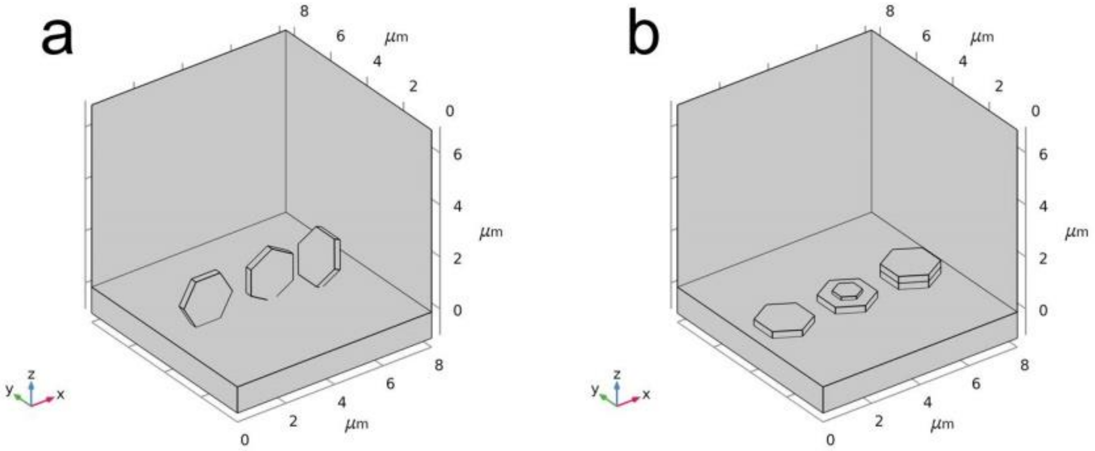

Figure S30. Schematic diagram of the COMSOL simulation model: a) Zn nanoplates on bare Zn; b) Zn nanoplates on NOC@Zn.

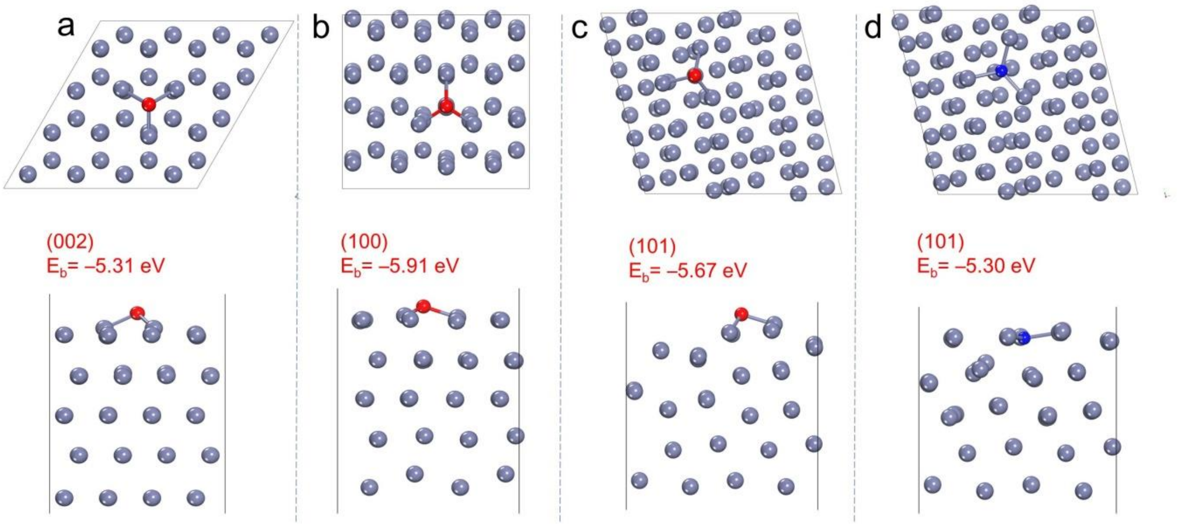

Figure S31. Optimized configurations with top (up) and side (down) view of the O adsorption on a) Zn (002) surface; b) Zn (100) surface; c) Zn (101) surface. d) Optimized N adsorption on Zn (101) surface. The gray, blue, and red balls denote the zinc, nitrogen and oxygen atoms, respectively.

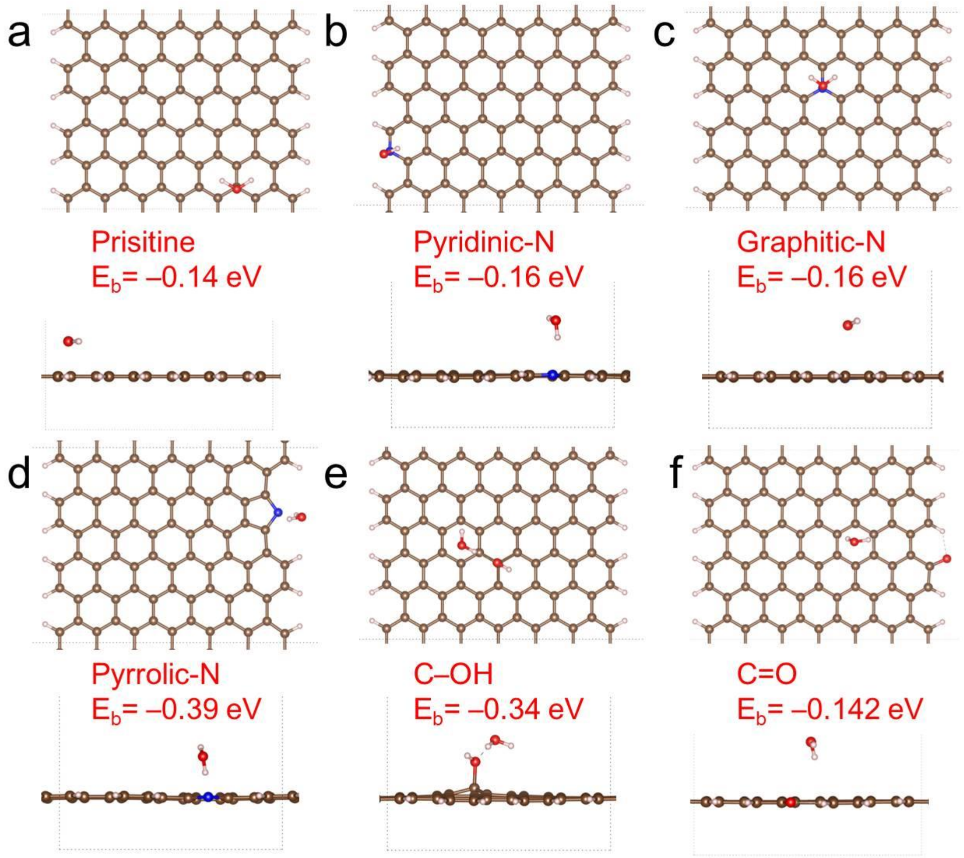

Figure S32. The adsorption configuration of H_2O molecule on NOC layer with different adsorption sites: a) prisitine; b) pyridinic-N; c) graphitic-N; d) pyrrolic-N; e) C–OH; f) C=O. The brown, blue, red and white balls denote the carbon, nitrogen, oxygen and hydrogen atoms, respectively.

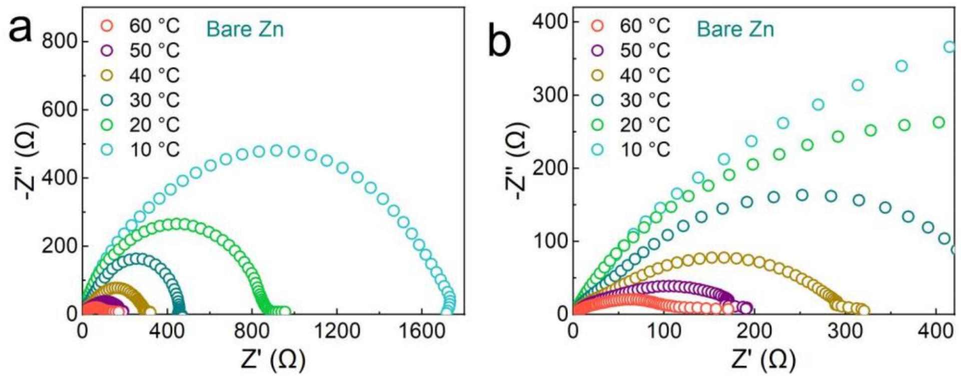

Figure S33. a) Nyquist plots at different temperatures for bare Zn. b) Enlarged view of a).

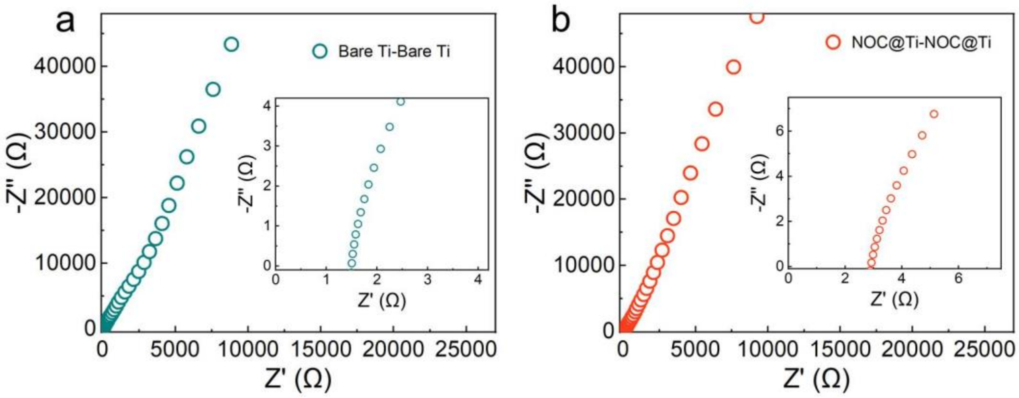

Figure S34. a) Nyquist plots for Ti–Ti symmetric cells. b) Nyquist plots for NOC@Ti–NOC@Ti symmetric cells. The resistance of NOC layer can be obtained from the Nyquist plots of Ti–Ti symmetric cells[1]. R (NOC) = R (total) – R (glass fiber) = 1.39 Ω. Thus the ionic conductivity of the NOC layer is ~6.9 $\times 10^{-6}$ S cm^{-1} by the following equation σ=L/(R*S), in which L is the thickness of the NOC layer (76 nm), S is the contact area (0.785 cm^2), and R = 1.39 Ω.

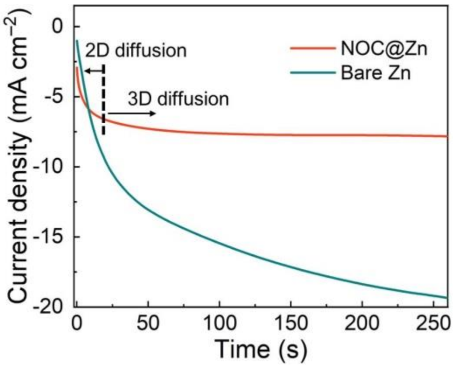

Figure S35. Chronoamperometry curves of NOC@Zn and bare Zn symmetric cells at a constant potential of –150 mV (2D: two-dimensional; 3D: three-dimensional).

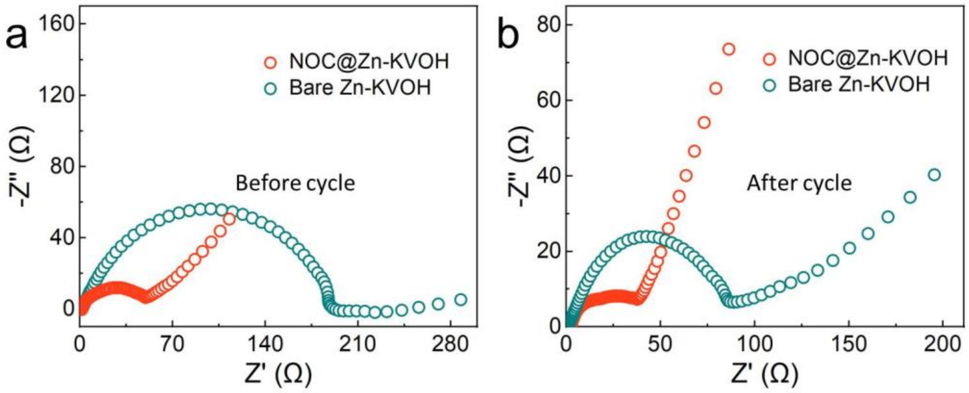

Figure S36. a) Nyquist plots of full cells before cycling. b) Nyquist plots of full cells after 100 cycles at 5 A g^{-1}.

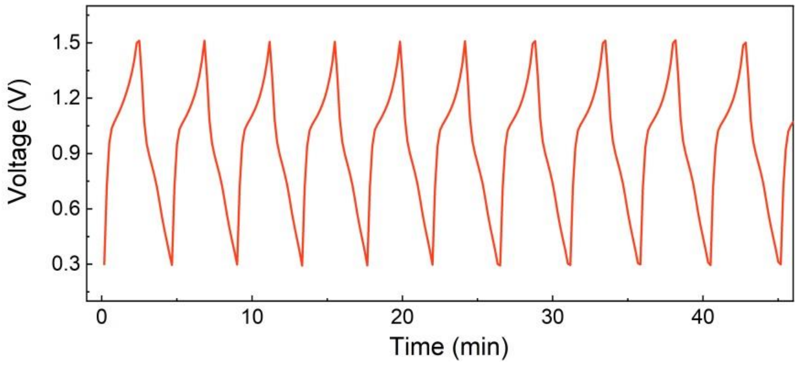

Figure S37. Detailed charge-discharge voltage profile of the NOC@Zn-KVOH full cell (Figure 5c) with a N/P ratio of 4.5.

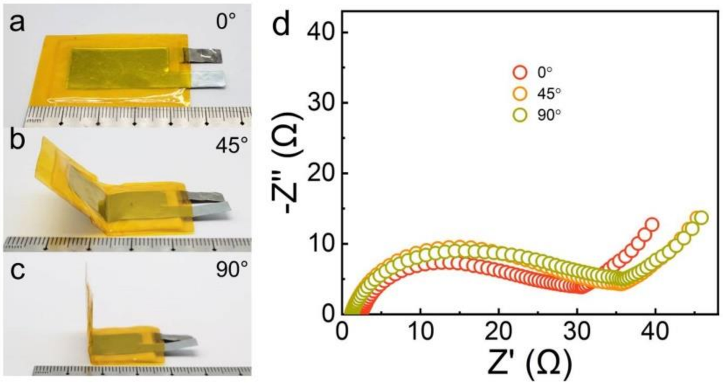

Figure S38. Digital images of flexible transparent NOC@Zn-KVOH cell under bending angles of: a) 0°, b) 45°, and c) 90°. d) Nyquist plots of NOC@Zn–KVOH cell under various bending angles.

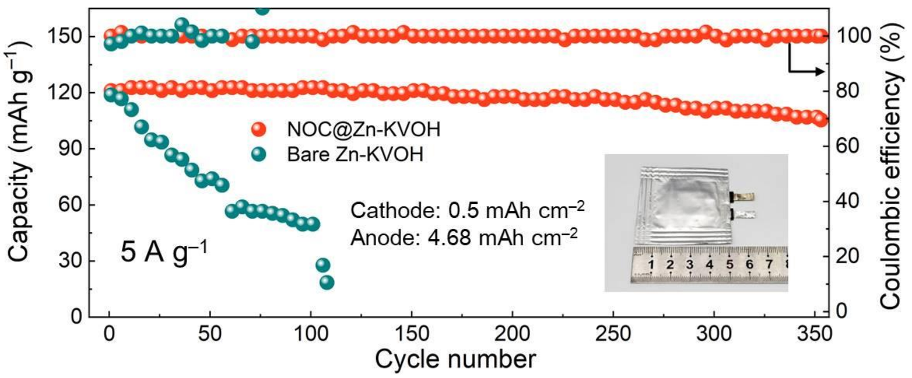

Figure S39. The long-term performance of the soft-packing battery with KVOH loading of 0.5 mAh cm^{-2} and Zn foil thickness of 8 μm (4.68 mAh cm^{-2}).

Supporting Table

Table S1. The conductivity of NOC layes with different growth rates. Here, the rise of pressure is used to indirectly indicate the growth rate.

**Growth rate** (Pressure rise)	5 Pa	8 Pa	10 Pa	40 Pa
**Conductivity** (S cm^{-1})	2.8×10^{-5}	2.0×10^{-5}	1.7×10^{-5}	$<5.0\times10^{-7}$

Table S2. Performance comparisons of reported Zn anodes protective strategies based on carbon materials.

Materials	Protecting methods	Preparation methods	Areal current (mA cm^{-2})	Areal capacity (mAh cm^{-2})	Voltage hysteresis (mV)	Lifespan (h)	Ref.
**NOC@Zn**	**Artificial Interphase layer (AIL)**	**PECVD**	**1**	**1**	**30**	**3040**	**This work**
rGO	AIL	Self-assembly	1	1	20	300	[11]
CNG	AIL	Acid hydrolysis method	1	0.5	21	2956	[13]
NGO	AIL	Langmuir–Blodgett method	1	1	17	1200	[14]
Graphite	AIL	Pencil drawing	0.1	0.1	28	200	[15]
CNTs	AIL	Dipping	0.5	0.15	20	600	[16]
$g\text{-}C_3N_4$	AIL	3D printing	1	1	55	1000	[17]
CNT foam	Host	CVD	3	0.5	143	167	[18]
CNT@CC	Host	CVD	2	2	27	200	[19]
Sn-PCF	Host	Electrospinning	1	1	22	750	[20]
GO	Separator	Vacuum filtration	5	-	100	500	[21]

NGDY	Separator	Cross-coupling reaction	5	1	40	500	[22]
Graphene	Separator	CVD	1	1	40	250	[23]
N-doped carbon	Separator	PECVD	1	1	27	1100	[24]

Table S3. Performance comparisons of dendrite-free Zn anodes protected by artificial interphase layer.

Materials	Areal current (mA cm^{-2})	Areal capacity (mAh cm^{-2})	Voltage hysteresis (mV)	Depth of discharge (DOD)	Lifespan (h)	Ref.
**NOC**	**1**<br>**30**<br>**20**	**1**<br>**30**<br>**36**	**30**<br>**100**<br>**75**	**1.7%**<br>**52.3%**<br>**63.0%**	**3040**<br>**136**<br>**60**	**This work**
ZnSe	1	1	16	1.9%	1530	[1]
CNG	10	10	100	17.1%	850	[13]
NGO	5	5	48	36%	300	[14]
ZIF-7	0.5	0.5	50	0.4%	3000	[25]
ZIF-8	2	1	29	4.4%	1200	[26]
Zn/Sn$_{(002)}$	0.5	1	8	1.2%	500	[27]
Gel-MA	2	2	130	6.8%	200	[28]
FCOF	5	3	50	5.1%	360	[29]
Sb	3	1	34	1.2%	1000	[30]
Nanoporous	0.25	0.05	52	0.2%	836	[32]
TiN	1	1	30	0.8%	2300	[32]
ZF@F-TiO_2	2	2	40	12.2%	280	[33]
ZnO	5	1.25	42	2.1%	500	[34]

ZnS	2	2	35	17.1%	1100	[35]
Mxene/ZnS	1	1	75	-	1100	[36]
ZrO_2	5	1	52	-	2100	[37]
ZnF_2	0.5	0.5	61	2.8%	700	[38]
Kaolin layer	4.4	1.1	70	1.9%	800	[39]
In	1	1	54	1.7%	520	[40]
Polyamide	0.5<br>10	0.25<br>10	100<br>80	-<br>85%	8000<br>150	[41]
Cyanoacrylate Adhesive	0.5<br>2	0.25<br>1	50<br>55	-<br>1.7%	800<br>400	[42]
ZnP	2<br>15	0.5<br>48	25<br>110	-<br>82%	3300<br>100	[43]

Table S4. Adsorption energy of the O/N atoms on different crystal planes encompassing Zn (002), Zn (100) and Zn (101).

**Adsorbed atom**	Zn (002)	Zn (100)	Zn (101)
O atom	–5.31 eV	–5.91 eV	–5.67 eV
N atom	–4.32 eV	–5.14 eV	–5.30 eV

Table S5. The fitting resistance results of symmetric cells for NOC@Zn and bare Zn at different temperatures.

**Symmetric cell**	**Resistance**	10 ℃	20 ℃	30 ℃	40 ℃	50 ℃	60 ℃
Bare Zn- Bare Zn	R_{ct} (Ω)	2220.6	829.8	350.5	300.5	150.4	99.6
NOC@Zn-NOC@Zn	R_{ct} (Ω)	61.7	42.0	30.4	25.9	18.5	15.8

Table S6. Summary and comparison of current density and areal capacity between this work and previous reports.

Cathode	Protection strategy	Areal current (mA cm^{-2})	Mass loading (mg cm^{-2})	Areal capacity (mAh cm^{-2})	Ref.
**KVOH**	**NOC@Zn**	**27.95**	**5.59**	**1.006**	**This work**
KVOH	ZnSe	5	1	0.194	[1]
NaVO	Sn-PCF	10	1	0.15	[20]
V_6O_{13}	N-Modified Graphdiyne	20.65	4.13	1.07	[22]
AC	Graphene	10	2	0.116	[23]
MnO_2	ZIF-7	2.94	4.2	0.76	[25]
MnO_2	TiN	1.48	2	0.46	[32]
MXene@ MnO_2	Mxene/ZnS	7.5	1.5	0.215	[36]
MnO_2	Al_2O_3	1	1.947	0.345	[44]
MnO_2	TiO_2/PVDF	0.616	1.000	0.244	[45]
VS_2	Polyzwitterionic hydrogel electrolyte	0.25	5	0.75	[46]
KB	Zn-BTC-modified separator	3.84	2	0.2	[47]
AC	ZIF-8-derived carbon	24	6	0.348	[48]
MnO_2	KPF_6 additives	5	1	0.15	[49]
Na:MnO_2/ GCF	Carbon film current collector	1.5	1.5	0.375	[50]

MnO_2	TiO_2	3	3	0.472	[51]
NaVO	Polyanionic hydrogel	6	1.2	0.198	[52]

Supporting Video

Video S1. *In situ* optical microscopy recording for bare Zn electrode during Zn plating process at 5 mA cm^{-2} (240 times faster).

Video S2. *In situ* optical microscopy recording for NOC@Zn electrode during Zn plating process at 5 mA cm^{-2} (240 times faster).

Supporting Reference

[1] X. Yang, C. Li, Z. Sun, S. Yang, Z. Shi, R. Huang, B. Liu, S. Li, Y. Wu, M. Wang, Y. Su, S. Dou, J. Sun, *Adv. Mater.* **2021**, *33*, 2105951.
[2] X. Yang, S. Lu, J. Peng, X. Hu, N. Wu, C. Wu, C. Zhang, Y. Huang, Y. Yu, H.-T. Wang, *Nano Res.* **2021**, *14*, 1103.
[3] G. Kresse, J. Hafner, *Phys. Rev. B* **1993**, *47*, 558.
[4] G. Kresse, J. Hafner, *Phys. Rev. B* **1994**, *49*, 14251.
[5] G. Kresse, J. Furthmuller, *Phys. Rev. B* **1996**, *54*, 11169.
[6] P. E. Blochl, *Phys. Rev. B* **1994**, *50*, 17953.
[7] J. P. Perdew, K. Burke, M. Ernzerhof, *Phys. Rev. Lett.* **1996**, *77*, 3865.
[8] H. J. Monkhorst, J. D. Pack, *Phys. Rev. B* **1976**, *13*, 5188.
[9] S. Grimme, J. Antony, S. Ehrlich, H. Krieg, *J. Chem. Phys.* **2010**, *132*, 154104
[10] J. Luo, L. Xu, H. Liu, Y. Wang, Q. Wang, Y. Shao, M. Wang, D. Yang, S. Li, L. Zhang, Z. Xia, T. Cheng, Y. Shao, Adv. Funct. Mater. 2022, 32, 2112151.
[11] A. Xia, X. Pu, Y. Tao, H. Liu, Y. Wang, *Appl. Surf. Sci.* **2019**, *481*, 852.
[12] J. Evans, C. A. Vincent, P. G. Bruce, *Polymer* **1987**, *28*, 2324.
[13] X. Zhang, J. Li, D. Liu, M. Liu, T. Zhou, K. Qi, L. Shi, Y. Zhu, Y. Qian, *Energy Environ. Sci.* **2021**, *14*, 3120.
[14] J. Zhou, M. Xie, F. Wu, Y. Mei, Y. Hao, R. Huang, G. Wei, A. Liu, L. Li, R. Chen, *Adv. Mater.* **2021**, *33*, 2101649.
[15] Z. Li, L. Wu, S. Dong, T. Xu, S. Li, Y. An, J. Jiang, X. Zhang, *Adv. Funct. Mater.* **2021**, *31*, 2006495.
[16] M. Li, Q. He, Z. Li, Q. Li, Y. Zhang, J. Meng, X. Liu, S. Li, B. Wu, L. Chen, Z. Liu, W. Luo, C. Han, L. Mai, *Adv. Energy Mater.* **2019**, *9*, 1901469.
[17] P. Liu, Z. Zhang, R. Hao, Y. Huang, W. Liu, Y. Tan, P. Li, J. Yan, K. Liu, *Chem. Eng. J.* **2021**, *403*, 126425.
[18] Y. Zhou, X. Wang, X. Shen, Y. Shi, C. Zhu, S. Zeng, H. Xu, P. Cao, Y. Wang, J. Di, Q. Li, *J. Mater. Chem. A* **2020**, *8*, 11719.

[19] Y. Zeng, X. Zhang, R. Qin, X. Liu, P. Fang, D. Zheng, Y. Tong, X. Lu, *Adv. Mater.* **2019**, *31*, 1903675.
[20] J.-L. Yang, P. Yang, W. Yan, J.-W. Zhao, H. J. Fan, *Energy Storage Mater.* **2022**, *51*, 259.
[21] J. Cao, D. Zhang, X. Zhang, M. Sawangphruk, J. Qin, R. Liu, *J. Mater. Chem. A* **2020**, *8*, 9331.
[22] Q. Yang, L. Li, T. Hussain, D. Wang, L. Hui, Y. Guo, G. Liang, X. Li, Z. Chen, Z. Huang, Y. Li, Y. Xue, Z. Zuo, J. Qiu, Y. Li, C. Zhi, *Angew. Chem. Int. Ed.* **2022**, *61*, e202112304.
[23] C. Li, Z. Sun, T. Yang, L. Yu, N. Wei, Z. Tian, J. Cai, J. Lv, Y. Shao, M. H. Rümmeli, J. Sun, Z. Liu, *Adv. Mater.* **2020**, *32*, 2003425.
[24] X. Yang, W. Li, J. Lv, G. Sun, Z. Shi, Y. Su, X. Lian, Y. Shao, A. Zhi, X. Tian, X. Bai, Z. Liu, J. Sun, *Nano Res.* **2022**, DOI: 10.1007/s12274-021-3957-z.
[25] H. Yang, Z. Chang, Y. Qiao, H. Deng, X. Mu, P. He, H. Zhou, *Angew. Chem. Int. Ed.* **2020**, *59*, 9377.
[26] X. Liu, F. Yang, W. Xu, Y. Zeng, J. He, X. Lu, *Adv. Sci.* **2020**, *7*, 2002173.
[27] S. Li, J. Fu, G. Miao, S. Wang, W. Zhao, Z. Wu, Y. Zhang, X. Yang, *Adv. Mater.* **2021**, *33*, 2008424.
[28] J. Shin, J. Lee, Y. Kim, Y. Park, M. Kim, J. W. Choi, *Adv. Energy Mater.* **2021**, *11*, 2100676.
[29] Z. Zhao, R. Wang, C. Peng, W. Chen, T. Wu, B. Hu, W. Weng, Y. Yao, J. Zeng, Z. Chen, P. Liu, Y. Liu, G. Li, J. Guo, H. Lu, Z. Guo, *Nat. Commun.* **2021**, *12*, 6606.
[30] L. Hong, L.-Y. Wang, Y. Wang, X. Wu, W. Huang, Y. Zhou, K.-X. Wang, J.-S. Chen, *Adv. Sci.* **2022**, *9*, 2104866.
[31] L. Kang, M. Cui, F. Jiang, Y. Gao, H. Luo, J. Liu, W. Liang, C. Zhi, *Adv. Energy Mater.* **2018**, *8*, 1801090.
[32] J. Zheng, Z. Cao, F. Ming, H. Liang, Z. Qi, W. Liu, C. Xia, C. Chen, L. Cavallo, Z. Wang, H. N. Alshareef, *ACS Energy Lett.* **2022**, *7*, 197.
[33] Q. Zhang, J. Luan, X. Huang, Q. Wang, D. Sun, Y. Tang, X. Ji, H. Wang, *Nat. Commun.* **2020**, *11*, 3961.
[34] X. Xie, S. Liang, J. Gao, S. Guo, J. Guo, C. Wang, G. Xu, X. Wu, G. Chen, J. Zhou, *Energy Environ. Sci.* **2020**, *13*, 503.
[35] J. Hao, B. Li, X. Li, X. Zeng, S. Zhang, F. Yang, S. Liu, D. Li, C. Wu, Z. Guo, *Adv. Mater.* **2020**, *32*, 2003021.
[36] Y. An, Y. Tian, C. Liu, S. Xiong, J. Feng, Y. Qian, *ACS Nano* **2021**, *15*, 15259.
[37] P. Liang, J. Yi, X. Liu, K. Wu, Z. Wang, J. Cui, Y. Liu, Y. Wang, Y. Xia, J. Zhang, *Adv. Funct. Mater.* **2020**, *30*, 1908528.
[38] J. Han, H. Euchner, M. Kuenzel, S. M. Hosseini, A. Groß, A. Varzi, S. Passerini, *ACS Energy Lett.* **2021**, *6*, 3063.
[39] C. Deng, X. Xie, J. Han, Y. Tang, J. Gao, C. Liu, X. Shi, J. Zhou, S. Liang, *Adv. Funct. Mater.* **2020**, *30*, 2000599.
[40] D. Han, S. Wu, S. Zhang, Y. Deng, C. Cui, L. Zhang, Y. Long, H. Li, Y. Tao, Z. Weng, Q. H. Yang, F. Kang, *Small* **2020**, *16*, 2001736.
[41] Z. Zhao, J. Zhao, Z. Hu, J. Li, J. Li, Y. Zhang, C. Wang, G. Cui, *Energy Environ. Sci.* **2019**, *12*, 1938.
[42] Z. Cao, X. Zhu, D. Xu, P. Dong, M. O. L. Chee, X. Li, K. Zhu, M. Ye, J. Shen, *Energy Storage Mater.* **2021**, *36*, 132.
[43] P. Cao, X. Zhou, A. Wei, Q. Meng, H. Ye, W. Liu, J. Tang, J. Yang, *Adv. Funct. Mater.* **2021**, *31*, 2100398.

[44] H. He, H. Tong, X. Song, X. Song, J. Liu, *J. Mater. Chem. A* **2020**, *8*, 7836.
[45] R. Zhao, Y. Yang, G. Liu, R. Zhu, J. Huang, Z. Chen, Z. Gao, X. Chen, L. Qie, *Adv. Funct. Mater.* **2021**, *31*, 2001867.
[46] K. Leng, G. Li, J. Guo, X. Zhang, A. Wang, X. Liu, J. Luo, *Adv. Funct. Mater.* **2020**, *30*, 2001317.
[47] H. Yang, Y. Qiao, Z. Chang, H. Deng, P. He, H. Zhou, *Adv. Mater.* **2020**, *32*, 2004240.
[48] Z. Wang, J. Huang, Z. Guo, X. Dong, Y. Liu, Y. Wang, Y. Xia, *Joule* **2019**, *3*, 1289.
[49] Y. Chu, S. Zhang, S. Wu, Z. Hu, G. Cui, J. Luo, *Energy Environ. Sci.* **2021**, *14*, 3609.
[50] Y. Wu, M. Wang, Y. Tao, K. Zhang, M. Cai, Y. Ding, X. Liu, T. Hayat, A. Alsaedi, S. Dai, *Adv. Funct. Mater.* **2020**, *30*, 1907120.
[51] K. Zhao, C. Wang, Y. Yu, M. Yan, Q. Wei, P. He, Y. Dong, Z. Zhang, X. Wang, L. Mai, *Adv. Mater. Interfaces* **2018**, *5*, 1800848.
[52] J. L. Yang, J. Li, J. W. Zhao, K. Liu, P. H. Yang, H. J. Fan, *Adv. Mater.* **2022**, *34*, 2202382.
